# Photorelease of phosphates: Mild methods for protecting phosphate derivatives

**DOI:** 10.3762/bjoc.10.212

**Published:** 2014-08-29

**Authors:** Sanjeewa N Senadheera, Abraham L Yousef, Richard S Givens

**Affiliations:** 1 Department of Chemistry, University of Kansas, Lawrence, Kansas 66045, U.S.A

**Keywords:** caged phosphates, hydroxynaphthylacetyl, organophosphorus, photo-Favorskii rearrangement, photoremovable protecting groups

## Abstract

We have developed a new photoremovable protecting group for caging phosphates in the near UV. Diethyl 2-(4-hydroxy-1-naphthyl)-2-oxoethyl phosphate (**14a**) quantitatively releases diethyl phosphate upon irradiation in aq MeOH or aq MeCN at 350 nm, with quantum efficiencies ranging from 0.021 to 0.067 depending on the solvent composition. The deprotection reactions originate from the triplet excited state, are robust under ambient conditions and can be carried on to 100% conversion. Similar results were found with diethyl 2-(4-methoxy-1-naphthyl)-2-oxoethyl phosphate (**14b**), although it was significantly less efficient compared with **14a**. A key step in the deprotection reaction in aq MeOH is considered to be a Favorskii rearrangement of the naphthyl ketone motif of **14a**,**b** to naphthylacetate esters **25** and **26**. Disruption of the ketone-naphthyl ring conjugation significantly shifts the photoproduct absorption away from the effective incident wavelength for decaging of **14**, driving the reaction to completion. The Favorskii rearrangement does not occur in aqueous acetonitrile although diethyl phosphate is released. Other substitution patterns on the naphthyl or quinolin-5-yl core, such as the 2,6-naphthyl **10** or 8-benzyloxyquinolin-5-yl **24** platforms, also do not rearrange by aryl migration upon photolysis and, therefore, do not proceed to completion. The 2,6-naphthyl ketone platform instead remains intact whereas the quinolin-5-yl ketone fragments to a much more complex, highly absorbing reaction mixture that competes for the incident light.

## Introduction

Phosphates have long held an important formative position in the development of organic photochemistry beginning with the seminal report by Havinga [1] of the unusual substituent effects in the photosolvolysis of aryl phosphates that showed unexpectedly pronounced *meta* activation of substituted aryl phosphates. A strong ‘*meta* effect’ resulting in enhanced reaction efficiency by electron withdrawing, *meta* substituents is contrary to their ground state effects on solvolysis reactions which, instead, display enhanced reactivity for *para* substituents. This contrasting substituent reactivity is a consequence of two different potential energy surface (PES) contours that control the reactivity, an observation first rationalized by Zimmerman through a comparison of the change in PES electron distribution in the ground (HOMO) and excited states (LUMO) using Hückel molecular orbital theory [2–3], subsequently attributed by him to the positioning of conical intersections between the HOMO–LUMO PE surfaces [4–5]. He termed this phenomenon the “*meta* effect”, and generalized it for photochemical solvolysis reactions. Several additional photosolvolysis studies substantiated the generality of the *meta* effect for phosphate leaving groups in heterolytic photoreactions.

**Figure 1:**
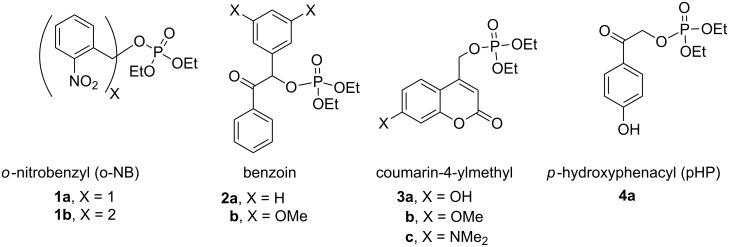
Common photoremovable protecting groups (PPGs) for phosphates depicted as diethyl phosphate (DEP) esters.

Over the past four decades, additional examples of phosphate esters attached to reactive chromophores other than phenyl and benzyl [6–11] have been investigated for their propensity to undergo heterolytic photosolvolysis reactions, most notably *o*-nitrobenzyl (o-NB) [12], benzyl phenyl ketone (benzoin) [13–14], coumarin-4-ylmethyl [15], and, more recently, *p*-hydroxyphenacyl (pHP) [15] phosphate esters (Figure 1). While these chromophores exhibit a range of photophysical properties, all share a conjugated aromatic structural motif that facilitates UV–vis absorption and serves as a traceless reagents, orthogonal to common ground state deprotection processes and essentially independent of pH effects. Thus, the chromophores are particularly useful at neutral pH and no added reagents are required for protecting group release.

Many photosolvolysis studies have targeted key biological substrates [6,8–12] for mechanistic and phenomenological studies. The culmination of these studies has resulted in the development of an entirely new branch of photochemistry with application in both organic chemistry and biochemistry. The reagents are generally described as “caged” compounds or photoremovable protecting groups (PPGs) in which the attached chromophore masks the biological activity of a substrate. Irradiation releases the substrate, allowing it to return to its normal bioactivity.

The desirable properties of any new PPG candidate are 1) absorption at wavelengths near or above 400 nm, 2) enhanced chemical and photochemical quantum yields and 3) improved rate of release, ideally in the range of ps to ns time constants; all three are useful properties for applications in organic chemistry and biochemistry, but they are particularly important for mechanistic studies in biochemistry and biophysics [6,8–11]. Most PPG candidates require sufficiently high energy excited states for heterolysis of a carbon–oxygen or carbon–heteroatom bond that binds the chromophore to a substrate. This limits the useful absorption range to ca. 350 to 400 nm for heterolysis by a primary photochemical pathway.

Phosphates hold a central historic position in caged photochemistry through their cross-disciplinary significance in both biology and chemistry. Nucleotides (especially ATP, cAMP, and GTP) were among the first to be covalently bound to chromophores in a cage or PPG format that were demonstrably released upon photolysis. Benzoin caged cAMP and *o*-nitrophenethyl caged ATP, seminal examples of caged phosphates, are often the two classic caged biochemical substrates cited [6–13,15–17]. Subsequent interest in and application of PPGs has exploded largely because they provide researchers in interdisciplinary fields with a tool for initiating biological [18] and chemical processes [6,8,10–11] by remote control with light as the activator. Spatial, temporal and concentration parameters for releasing substrates are controlled by adjusting the focus, time resolution, and intensity of the light source in conjunction with other variables such as the photochemical reactivity and molecular reaction pathway. Since photolysis reactions are generally considered to be kinetically much faster than ground state processes (most photorelease rates have sub μsec time constants), the use of time resolved lasers to activate PPGs offers much greater temporal resolution and more precise spatial control of the reaction variables in chemical processes, increasingly important features for biophysics and biochemistry.

Our studies on photosolvolysis reactions have uncovered several new chromophores with superior PPG properties amenable to photodeprotection. Earlier work defined the advantages and limitations of *p*-hydroxyphenacyl (pHP, **4**), a PPG that is now finding application in biochemistry and chemistry [15]. We now report new pHP analogs with a fused aromatic or heteroaromatic extension of pHP. Our intent was to impose the pHP motif on the naphthyl and indolin-5-yl platforms (maintaining the critical substituent pattern) in order to foster aryl ketone migration by a photo-Favorskii rearrangement concomitant with ligand release. The combination of these features shifts and extends the chromophore, exhibiting a more intense, broader absorption band closer to the visible region, making the PPG more accessible for photodeprotection. Thus, elaborating the two motifs by optimally appending a hydroxy and carbonyl positioned for excited state interaction, in accord with the pHP motif **4a**, invited serious examination.

## Results

### Synthesis of phosphate esters

**Scheme 1:**
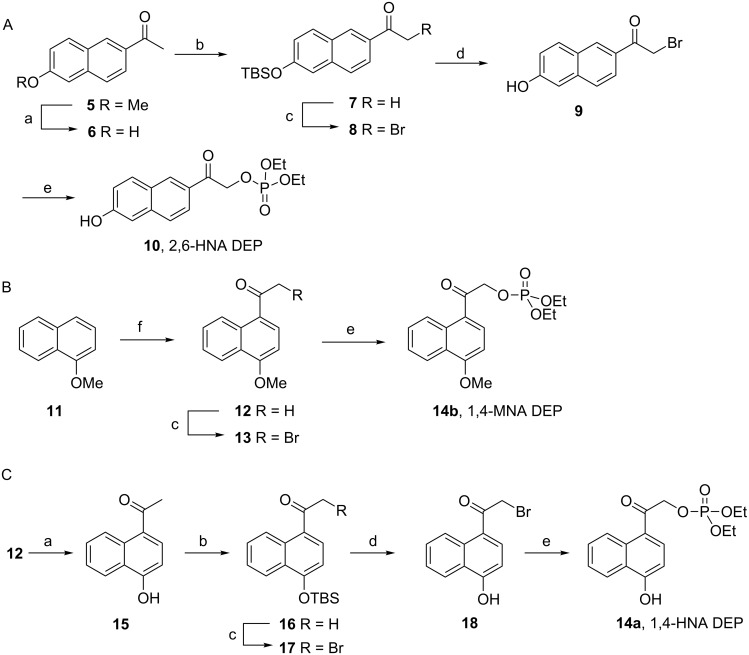
Synthesis of 2,6-HNA DEP (**10**), 1,4-HNA DEP (**14a**), and 1,4-MNA DEP (**14b**) DEP esters. Reagents and conditions: a) PhSH, K_2_CO_3_, *N*-methyl-2-pyrrolidinone, 194 °C, 45 min; b) TBSCl, Et_3_N, DBU, CH_2_Cl_2_, rt, 28 h; c) CuBr_2_, 1:1 CHCl_3_/EtOAc; d) KHSO_4_, MeOH (aq), CH_2_Cl_2_, rt, 43 h; e) tetramethylammonium diethyl phosphate, DMF, 55 °C, 2–3 h; f) Ac_2_O, AlCl_3_, CH_2_Cl_2,_ 0 °C to rt over 3 h then stirred at rt.

**Scheme 2:**
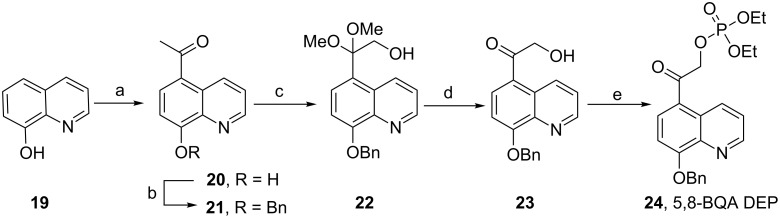
Synthesis of diethyl 8-(benzyloxy)quinolin-5-yl)-2-oxoethyl phosphate (5,8-BQA DEP, **24**). Reagents and conditions: (a) CH_3_COCl, AlCl_3_, nitrobenzene, 14 h, 40%; (b) PhCH_2_Br, K_2_CO_3_, DMF, rt, 24 h, 100%; (c) PhI(OAc)_2_, MeOH, KOH, 0 °C to rt, 24 h, 90%; (d) 50% CH_3_COOH, rt, 12 h, 98%; (e) P(O)Cl(OEt)_2_, pyridine, −5 °C to rt, 24 h, 30%.

A series of diethyl phosphate (DEP) esters caged with extended 2-(6-hydroxynaphthalen-2-yl)-2-oxoethyl (2,6-HNA, **10**), 2-(4-hydroxynaphthalen-1-yl)-2-oxoethyl (1,4-HNA, **14a**) and its methoxy ether (1,4-MNA, **14b**)**,** and 8-(benzyloxy)quinolin-5-yl)-2-oxoethyl (5,8-BQA, **24**), each modeled after the *p*-hydroxyphenacyl (pHP, **4**) chromophore were synthesized as illustrated in Scheme 1 and Scheme 2.

Synthesis of the extended 2,6-HNA phosphate ester **10** was accomplished by first demethylating 2-acetyl-6-methoxynaphthalene (**5**) with thiophenol and 0.1 mol percent potassium carbonate in *N*-methyl-2-pyrrolidinone at 194 °C [19] to generate the hydroxynaphthalene **6** (72%) followed by protecting the hydroxy group with TBSCl to give **7** (89%, Scheme 1A). α-Bromination with copper(II) bromide [20] gave **8** (96%, direct bromination of **6** gave poor yields). The TBS group was removed in 30% aqueous methanol containing 10–20% methylene chloride and several molar equivalents of potassium hydrogen sulfate to provide hydroxy bromo ketone **9** (yield, 75%) [21], which was reacted with tetramethylammonium diethyl phosphate under anhydrous conditions to afford diethyl (2-(6-hydroxynaphthalen-2-yl)-2-oxoethyl) phosphate (2,6-HNA DEP, **10**, 62%) [22].

Diethyl (2-(4-methoxynaphthalen-1-yl)-2-oxoethyl) phosphate (1,4-MNA DEP, **14b**) was obtained, as shown in Scheme 1B, beginning with Friedel–Crafts acylation of 1-methoxynaphthalene (**11**) yielding 1-acetyl-4-methoxynaphthalene (**12**, 73%). α-Bromination with copper(II) bromide gave **13** (44%) which was converted to the phosphate ester using tetramethylammonium diethyl phosphate in dimethoxyethane (DME) at room temperature to give diethyl (2-(4-methoxynaphthalen-1-yl)-2-oxoethyl) phosphate (1,4-MNA DEP, **14b**, 92%).

The hydroxy analog (1,4-HNA DEP, **14a**, Scheme 1C), was synthesized from methoxynaphthone **12** by demethylation with thiophenol giving acetylnaphthol **15** in moderate yield (42%). Protection with TBSCl gave **16** (86%) followed by α-bromination to **17** (89%). TBS deprotection gave 2-bromo-1-(4-hydroxy-1-naphthyl)ethanone, (**18**, 59%) which was converted to diethyl (2-(4-hydroxynaphthalen-1-yl)-2-oxoethyl) phosphate (1,4-HNA DEP, **14a**, 31%) with tetramethylammonium diethyl phosphate.

Benzyl protected diethyl 2-(8-hydroxyquinolin-5-yl)-2-oxoethyl phosphate (**24**) was synthesized from 8-hydroxyquinoline (**19**) by first performing acylation [23] with acetyl chloride and then quantitatively protecting the hydroxy group as its benzyl ether [24] followed by installation of ketal protection with iodobenzene diacetate in alkaline methanol [25] to give **22** (90%, Scheme 2). Deprotection of **22** with 50% acetic acid gave the α-hydroxy ketone **23** (98%) which was esterified with diethyl phosphoryl chloride in pyridine [26] to afford 2-(8-(benzyloxy)quinolin-5-yl)-2-oxoethyl diethyl phosphate (5,8-BQA DEP, **24**, 30%).

**Figure 2:**
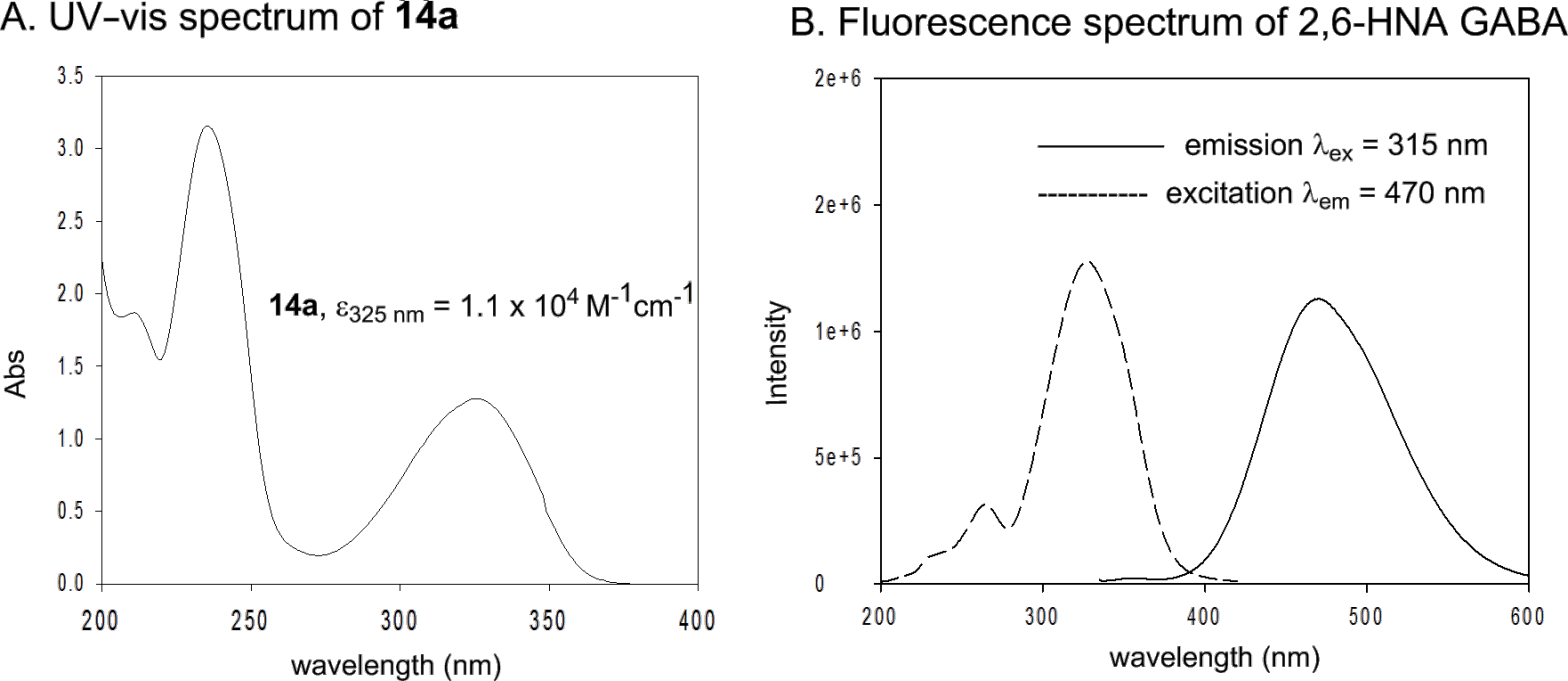
A. UV–vis spectrum of **14a** (1,4-HNA DEP) in 1% aq MeCN. B. Fluorescence emission/excitation spectra of 2,6-HNA GABA (0.042 mM) in pH 7.3 TRIS buffer containing 1% MeCN.

The UV–vis spectra of 1,4-substituted esters **14a,b** (λ_max_ range from 319 to 325 nm) are substantially red-shifted relative to the pHP ester **4a** (λ_max_ at 287 nm) and exhibit molar extinction coefficients (ε) of ca. 10^4^ M^−1^cm^−1^ in aq MeCN (Figure 2). For the 2,6-HNA DEP (**10**) and 2,6-HNA GABA, the λ_max_ appears at 325 nm whereas the λ_max_ for the 5,8-BQA phosphate **24** occurs at 321 nm in 10% aq MeCN. For the 2,6-HNA series, a strong fluorescence emission is observed at 470 nm as shown here for the more aqueous soluble GABA ester derivative. The 2,6-HNA GABA had better aqueous solubility for fluorescence studies. (See Supporting Information File 1 for synthetic and spectral details).

### Photolysis of phosphate esters

**Scheme 3:**
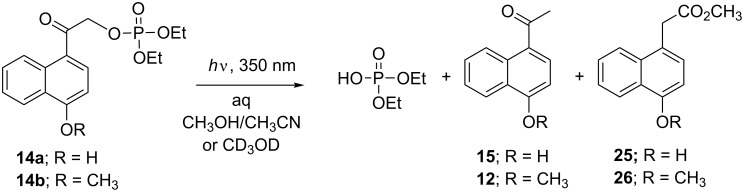
Photolysis of 1,4-HNA and 1,4-MNA diethyl phosphates **14a** and **14b** in aq MeOH.

**Scheme 4:**
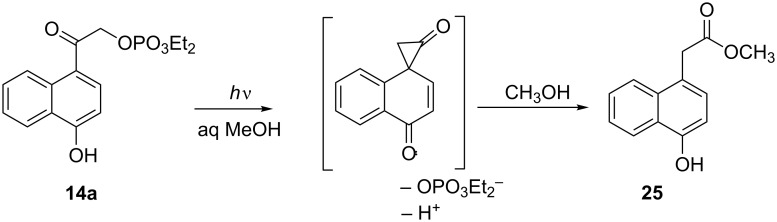
The photo-Favorskii rearrangement of **14a**.

Irradiation of **14a** at 350 nm in Pyrex vessels under ambient conditions in 1% aq CD_3_OD resulted in the release of diethyl phosphate as confirmed by NMR (Scheme 3, Table 1). The protecting group underwent a photo-Favorskii rearrangement (Scheme 4), yielding predominantly methyl 4-hydroxy-1-naphthylacetate (**25**) with a trace amount of reduction product **15** (Scheme 3). In anhydrous methanol, the ratio of **25**/**15** was approximately 3:1. When the photolysis was performed in 10% aq MeCN, **15** was the only assigned structure from the ^1^H NMR spectrum of a complex product mixture.

**Table 1:** Photolysis efficiencies and product yields for 1,4-HNA DEP (**14a**) and 1,4-MNA DEP (**14b**).

ester	% conversion^a^	deprotection % yield^b^	Φ_dis_^c^

**14a** OH	98	100	0.028
**14b** OMe	40	90	0.0076

^a^One hour photolysis in CD_3_OD at 350 nm, determined by ^1^H NMR using DMF as an internal standard, ^b^diethyl phosphate, corrected for conversion, ^c^determined by RP-HPLC; photolysis in 1% aqueous MeOH.

Photolysis of the methoxy analog **14b** under similar conditions either in CD_3_OD or 1% aq MeCN resulted in the release of DEP and formation of **12** as confirmed by reversed-phase HPLC (RP-HPLC) and NMR (Scheme 3, Table 1) but the conversion was not complete and the reaction was lethargic due to competitive absorption by the unrearranged product chromophore **12**. In 1% aq MeOH, however, the major product from the protecting group was methyl 4-methoxy-1-naphthylacetate (**26**) which was characterized by the signals at δ 4.02 and 3.68 ppm and confirmed by FAB–MS (*m*/*z* 230) and comparison with an independently synthesized sample obtained from Friedel–Crafts alkylation of 1-methoxynaphthalene (**11**) with methyl bromoacetate and ferric oxide [27].

Quantum yields for disappearance and percentage conversions for **14a** are higher than that of **14b** as indicated in Table 1. After one hour of photolysis at 350 nm in CD_3_OD, **14a** reached 98% conversion whereas **14b** managed to attain only 40% conversion. The disappearance quantum efficiency (Φ_dis_) for **14a** in 1% aq CD_3_OD was 0.028, more than three times that for **14b** under the same conditions. However, the deprotection yields for both esters were excellent, resulting in quantitative release of DEP based on conversion.

The quantitative conversion and apparent good efficiency of the 1,4-derivative **14a** prompted further investigation. The quantum yield for disappearance of **14a** in 1% aq MeCN (Φ_dis_ = 0.021) is comparable to that observed in aq MeOH (Φ_dis_ = 0.028, Table 1 and Table 2) but the product mixture was more complex (vide infra). Interestingly, quantum yields for the disappearance for **14a** are enhanced three-fold (Φ_dis_ = 0.067) when the photolysis solution was purged free of oxygen (Table 2). The quantum yield of **14a** for the triplet sensitized photolysis in the presence of benzophenone (BP), a well-established triplet sensitizer, at 254 nm under O_2_-free conditions was Φ_dis_ = 0.022. These combined results demonstrate that the reactive excited state is a triplet that is partially quenched by O_2_ under ambient conditions.

**Table 2:** Photolysis of 1,4-HNA diethyl phosphate (**14a**) in the presence and absence of O_2_ and benzophenone in 1% aq MeCN.

Φ_dis_^a^	conditions

0.067^b^	degassed with Ar
0.022	BP sensitized^c^
0.021	saturated with Air

^a^Determined with RP-HPLC; ^b^Estimated value (see experimental section); ^c^Benzophenone (11 mM) as the sensitizer and **14a** (0.59 mM) in 1% aq MeCN at 254 nm and purged with Ar.

**Scheme 5:**
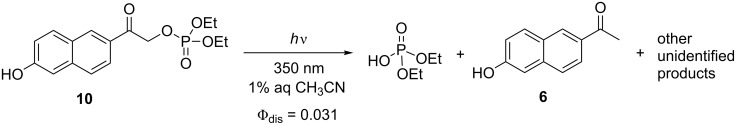
Photolysis of 2,6-HNA DEP (**10**) in 1% aq MeCN.

Irradiation of 2,6-HNA DEP (**10**) at 350 nm in 1% aq MeCN under conditions comparable to those employed with **14a**,**b** also released phosphate. The disappearance quantum yield for **10** (Φ_dis_ = 0.031) was nearly the same as that obtained for **14a** but the conversion after a 10 min irradiation was only 67%. Unlike **14a**,**b**, however, the chromophore did not undergo the photo-Favorskii rearrangement, forming only the reduction product 2-acetyl-6-hydroxynaphthalene (**6**) in addition to other unidentified photoproducts (Scheme 5). These results paralleled our observations for **14a**,**b** in aq MeCN.

**Scheme 6:**
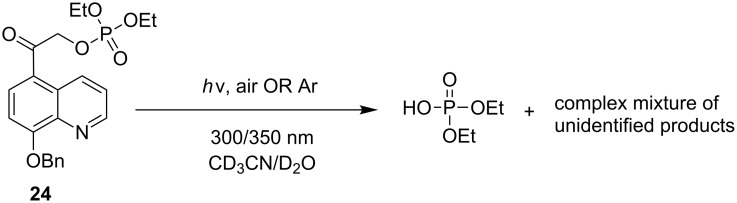
Photolysis of 5,8-BQA diethyl phosphate (**24**).

Finally, photolysis of 5,8-BQA diethyl phosphate (**24**) in 10% aq MeCN at 300 or at 350 nm under degassed conditions released DEP at only 30% conversion, even after 18 hours as confirmed by both ^1^H and ^31^P NMR analyses (Scheme 6). The quantum yields for disappearance and product appearance (Φ_dis_ = Φ_app_ = 0.00024) were exceptionally low (Table 3), considerably less than any of the naphthyl (**10** and **14**) or phenacyl (**4a**) analogs (see Table 4, Discussion section). A complex product mixture of byproducts of the indolinyl chromophore was obtained which was not investigated further.

**Table 3:** Quantum yields and conversions of **24** with and without Ar purging.^a^

ester	% conversion^b^ ambient O_2_	% conversion^b^ Ar purged	Φ_dis_ (× 10^3^)^c^ ambient O_2_	Φ_app_ (× 10^3^)^d^ ambient O_2_

**24**	32	30	0.24	0.24

^a^Solvent was 10% H_2_O in MeCN. Photolyses were conducted with 300 or 350 nm lamps. ^b^Determined using ^1^H NMR in 10% D_2_O in CD_3_CN; ^c^Determined with ^1^H NMR for the disappearance of the starting material at 350 nm with air; ^d^Determined with ^1^H NMR for the appearance of diethyl phosphate at 350 nm with air.

## Discussion

In our design of new PPG chromophores, we have attempted to circumvent or avoid the inherent deficiencies of the classical o-NB, benzoin, and coumaryl analogs (see Figure 1). A noisome limitation of o-NB PPGs that mitigate their general use in biological and mechanistic studies are their inherently slow reaction rates. o-NB rate constants are decidedly among the slowest for a photochemical heterolysis (e.g., usually not larger than μsec time constants) due to mechanistic constraints imposed by the rate determining step of a ground state hemiacetal hydrolysis, generated photochemically, that releases the substrate. Furthermore, the o-NB PPG chromophore, itself, is converted to an *o*-nitroso aldehyde (or ketone) that reacts with endogenous nucleophiles such as amines, thiols, etc. either present on the substrate, on target proteins or in the media. Poignant evidence of these limitations surfaced during mechanistic investigations of GAP protein GTPases and enzymatic studies of ATPase [28–34].

We [15,28–29,35] and others [6–11] have successfully offered alternatives to the o-NB PPG series, initially with benzoin phosphate **2** [13–14] and coumarin-4-ylmethyl phosphate **3**. However, the chromophores for these candidates remain intact or rearrange to more strongly absorbing chromophores which compete for the effective radiation wavelengths, often resulting in incomplete conversions that compromise their synthetic utility. These chromophores and especially their photoproducts are also highly fluorescent, which frequently prove to be disadvantageous.

**Figure 3:**
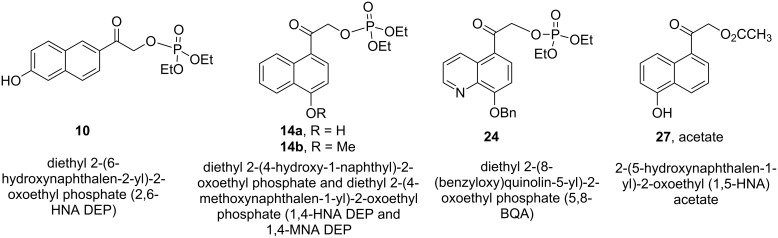
Naphthyl and quinolin-5-yl caged phosphate esters **10**, **14**, **24** and **27** (acetate ester).

The recent candidates are based on a pHP motif. Phosphate esters of **10**, **14**, **24**, and acetate **27** [19] each possess a hydroxy donor coupled with an acetyl function bearing the leaving group (Figure 3). One of these, the 5-hydroxy-1-naphthylacetyl motif as its acetate ester **27**, has already been reported to be photochemically inert in 1:1 aq MeCN by Wan [19]. Accordingly, we did not pursue the study of **27** (vide infra). Extending the aromatic portion of the chromophore with either an added benzo or pyridyl group using the pHP model red-shifted the chromophore absorption for the remaining three, **10**, **14**, and **24** (e.g., the maximum of **4a** at 280 nm shifted to 325 nm for **14a** tailing nearly to 400 nm, see Figure 2).

**Figure 4:**
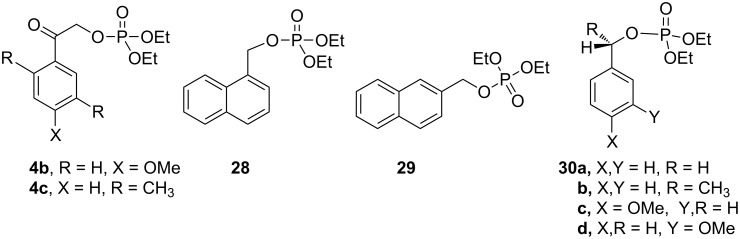
Previously studied caged diethyl phosphate PPGs possessing aromatic (benzyl, phenacyl, and naphthylmethyl) phosphates.

Similar extensions of the benzyl PPG expanding it to naphthylmethyl [35–36] (**28** and **29**, Figure 4) or modifications by attaching substituents to phenacyl (e.g., **4b,c**) or benzyl (**30b**–**d**) moieties, and naphthylacetyl [37] reportedly lowered the singlet and triplet state energies only modestly [10–11] and were unsuitable because these chromophores remained intact and in competition for incident radiation.

Photolysis of 1,4-HNA DEP (**14a**) in 1% aq MeOH released DEP with modest efficiency, Φ = 0.028. Similarly, the methoxy analog, 1,4-MNA DEP (**14b**), released DEP, but with a much lower efficiency (Φ = 0.0078). Both quantitatively produced DEP and, pleasingly, underwent photo-Favorskii rearrangements converting the aryl ketones to methyl naphthylacetates, blue-shifting the byproduct chromophore absorptions.

Both **14a** and **14b** now become the first new examples of photo-Favorskii rearrangements, here on a naphthyl platform. Previous examples are limited to phenacyl frameworks [6–9,15,38–39]. It is interesting to note, however, that replacement of the OH with OMe in the 1,4-HNA series results in both lower efficiency and decreased yield, a further analogy to that found with *p-*methoxy vs *p*-hydroxyphenacyl DEPs **4a,b** (Table 4) [6,13,15,40] and with other leaving groups.

In contrast to the results in 1% aq MeOH, photolysis of **14a**,**b** in anhydrous media or changing the co-solvent to MeCN resulted in a decrease in conversion (40–60%) and more complex reaction mixtures. The only products identified were DEP (quantitative) and reduction products **12** or **15**, signaling a change in mechanism from heterolysis to homolysis. The low conversions are primarily due to competition for incident radiation by the unrearranged byproduct chromophore **12** or **15**.

**Scheme 7:**
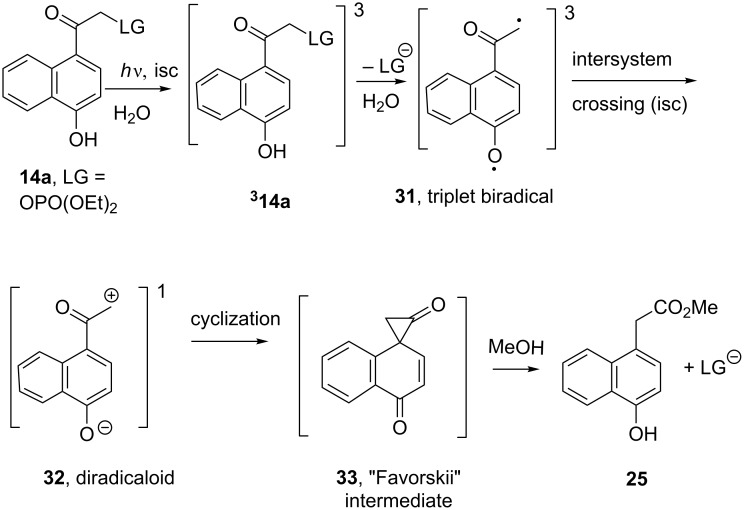
Photo-Favorskii mechanism based on pHP DEP **4a** photochemistry as applied to 1,4-HNA DEP (**14a**).

The role of H_2_O and hydroxylic solvents observed in this series is in complete accord with solvent effects on the parent pHP photoreactions [6,13,15,40–46]. The photo-Favorskii reaction (Scheme 7) is favored by aqueous-based solvents that serve both as a proton donor and an acceptor to the conjugate base generated from the chromophore’s triplet state heterolytic cleavage of the leaving group. The dual behavior of H_2_O is manifest in accepting a proton from the naphthol OH while simultaneously solvating the developing anionic charge on the departing nucleofuge. The resulting biradical **31**, which must be a triplet by Wigner’s spin rule [47] is formed from the chromophore while on the excited state PES. Intersystem crossing (isc) to a diradicaloid ground state intermediate **32** (vide infra) is followed by cyclization to form the ‘Favorskii intermediate’ **33** [6,8,15,26,38–39,48].

In contrast to the 1,4-naphthyl design, neither **10** nor **24** underwent Favorskii rearrangements. While both gave stoichiometric release of DEP, the chromophores either remain intact (for **10**) as also reported for other 2-naphthylacetyl analogs [49] or are consumed by oxidation and fragmentation as in the case of **24**. Thus, the conversion and chemical yields were low and the photolysates were complex mixtures of byproducts. In the case of **24**, although the reaction efficiency was insensitive to O_2_ (implying singlet reactivity, not quenchable by ambient O_2_), the product mixture included several oxidation byproducts of the chromophore. Thus, photoreactions of **24** with ambient O_2_ were a complication not encountered with **10** or **14a**,**b**.

The failure to deconjugate the carbonyl of either chromophore by rearrangement once again impinged on the overall conversion to released substrate. The formation of competing chromophoric byproducts limits the conversion and yields [6,10–11,15]. For example, changing the leaving group for **10**, i.e., photolysis of 6-hydroxy-2-naphthylacetyl GABA ester (Figure 2B) inefficiently released 4-aminobutyrate (GABA) in low yield and is absent of any chromophore rearrangement.

It is surprising that 2,6-HNA DEP (**10**) and Wan’s 1,5-HNA acetate **27** [19] do not undergo a photo-Favorskii rearrangement. In the case of **27**, Wan suggested that the lack of reactivity may have arisen from factors such as a lower excited state energy of the naphthyl ketone platform or the disruption of both aromatic rings for the 1,5 substituted analog **27** whereas only one ring is involved for the 1,4 substituted derivative [19].

Both of the contributing components of the 2,6 pattern, 2-naphthol (*E*_T_ = 60.2 kcal/mol) and 2-acetylnaphthalene (*E*_T_ = 59.5 kcal/mol) are higher energy contributors than the same two components of the 1,4 and 1,5 patterns, 1-naphthol (*E*_T_ = 58.6 kcal/mol) and 1-acetylnaphthalene (*E*_T_ = 56.4 kcal/mol) [10–11]. Of the two motifs, the 1,4 arrangement has the lower energy triplet. Yet the 2,6 pattern with the higher triplet energy is the less reactive and fails to participate in the photo-Favorskii rearrangement.

**Scheme 8:**
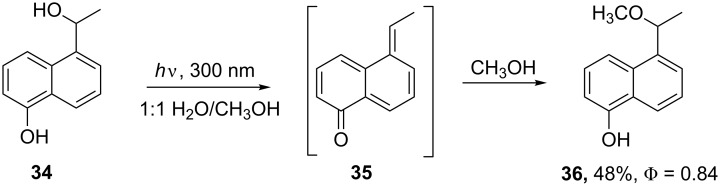
Photodehydration and substitution of 5-(1-hydroxyethyl)-1-naphthol **34** [19].

Wan’s work clearly demonstrates, however, that the excited state acidity of 1-naphthols is much greater than 2-naphthols and that excited-state intramolecular proton transfer (ESIPT) for 1-naphthols occurs at both the 5- and 8-positions by H–D exchange [19]. Furthermore, dehydration of 5-(1-hydroxyethyl)-1-naphthol (**34**) is very efficient, leading to quinone methide **35** which is trapped by solvent MeOH (Scheme 8). The corresponding 4-(1-hydroxyethyl)-1-naphthol, however, is unreactive. Comparisons of the two photoreactions, photodehydration and photo-Favorskii [48,50], and Wan’s results [19] for H–D exchange reactions demonstrate the importance of the relative location of the two functional groups and the role of ESIPT for both reactions. The hydroxy group, as Wan has elegantly determined, is the source of the acidic proton (especially for singlet state reactions). The carbonyl appendage, however, must be responsible for the enhanced heterolysis efficiency [28–29,38] and may influence the formation of the triplet excited state through intersystem crossing (isc) [26] which is the origin of the heterolysis and rearrangement processes for pHP.

**Scheme 9:**
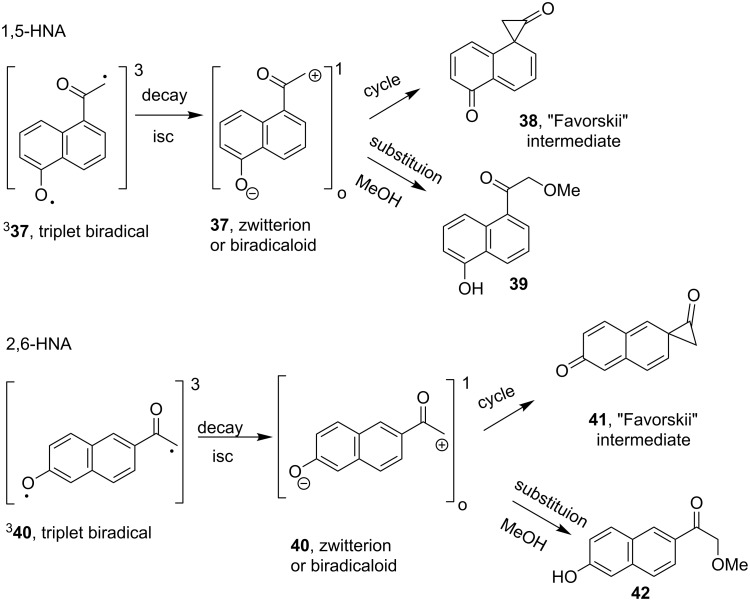
Putative rearrangement intermediates for 1,5- and 2,6- HNA chromophores.

Wan’s alternative suggestion that a disruption of both aromatic rings' π-conjugation would be less favorable than only one ring’s disruption has merit. For this to be a determining factor, the disruption would have to occur prior to or during the product determining formation of the ‘Favorskii’ intermediate **33** [38,48,50]. The putative key intermediates for 2,6-HNA and 1,5-HNA photo-Favorskii rearrangements (Scheme 9) illustrate the stage at which disruption in the π-network takes place. As in the case for the 1,4-HNA rearrangement, neither triplet biradical ^3^**37** nor ^3^**40** experience a change in connectivity within the aromatic nucleus. The formation of the triplet biradical is irreversible so that once generated, it must proceed on to a final product. Since no rearrangement products are formed for either the 2,6-HNA or 1,5-HNA, it is tempting to conclude that the decay to a ground state biradicaloid or zwitterion is the product determining step. In both cases, the aromaticity of both rings is lost upon formation of the Favorskii cyclopropanone intermediates **38** and **41**.

However, this rationale also falls short because the reactions of the similar biradicaloid or zwitterion are known to undergo nucleophilic substitution and not reduction to the methyl ketone products **6**, **12** or **15** [38–39]. Substitution by solvent would trap the intermediate biradicaloid (or zwitterion) to form, in the case of 2,6-HNA, the α-methoxy ketone **42**, which was not detected among the photoproducts.

The controlling feature leading to the change in mechanism for these two examples is more reasonably imbedded in the route to the reactive triplet excited state configuration [10–11]. For pHP, the reactive excited state is a π,π* triplet that is favored only in hydroxylic solvents, especially H_2_O [15,45–46,48]. In non-hydroxylic solvents, the (n,π*)^3^ dominates which leads to the classical α- and β-homolytic cleavage and H-abstraction reactions. For 2,6-HNA and 5,8-BQA, the reduction product, a methyl ketone, probably results from homolysis. The lower excited state acidity and the poor intersystem crossing in the 2,6-HNA platform and strong fluorescence of the 2,6-HNA chromophore (Figure 2) also diminishes formation of the triplet and, thus, its participation in a photo-Favorskii rearrangement. Finally, an indication of the photostability of the 2,6-HNA derivatives had earlier precedence in the lack of product formation for 2-hydroxy-6-trifluoromethylnaphthylene photo-dehydrofluorination reported by Seiler and Wirz [51]. For the 1,5-HNA acetate, the less reactive (higher p*K_a_*) of the leaving OAc group of **27** vs DEP, fluorescence decay and reversible ESIPT processes are major factors disfavoring photorelease.

A summary of the most frequently encountered examples of caged phosphates is given in Table 4. The data are reported for DEP leaving groups, when available, since DEP has proven to be a commonly employed test leaving group for PPG comparisons. It is generally found that the DEP model is reliable when extended to more complex phosphate leaving groups including nucleotides such as ATP and GTP and tyrosyl phosphates and thiophosphates [52–55]. The key comparisons for practical applications of photochemical deprotection are the maximum conversion which controls the chemical yield, the quantum yield (or photochemical efficiency) and the complexity of the photochemical products and byproducts in the photolyzate mixture. Solvents and excitation wavelengths are also given in Table 4 as a guide on selecting a PPG using these reaction parameters.

**Table 4:** A comparison of photorelease from cages for diethyl phosphates. Chemical yields, quantum yields, efficiencies and complexity based on solvents and chromophores.

PPG chromophore	derivatives	solvent^a^	λ_exc_ nm	Φ_dis_ and (conversion yield/%)	S^1^ or T^1^	chromo- phore fate^b^	Ref

*o*-nitrobenzyl 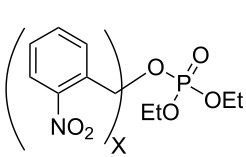	**1a** X=1	H_2_O	254, 308	N/A^c^ (28^d^)		mixture	[56]
	**1b** X=2	H_2_O	308, 355	N/A (90^d^)	S^1^	N/A	[56]
benzyl 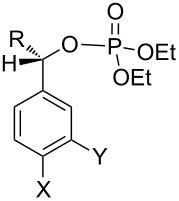	**30a** (R, X, Y = H)	MeOH, *n*-BuOH, *t*-BuOH	254	0.39 (50–60)	S^1^	intact	[35,57]
	S(−)-**30b** (X, Y = H, R = Me)	MeOH *t*-BuOH	254	0.21–0.9^e^ (77/28 rac)	S^1^	intact	[35,58]
	**30c** (R, Y = H, X = OMe)	*n*-BuOH, *t*-BuOH	254	0.42 (47)	S^1^	intact	[58]
	**30d** (R, X = H, Y = OMe)	*t*-BuOH	254	0.18 (28)	S^1^	intact	[58]
phenacyl 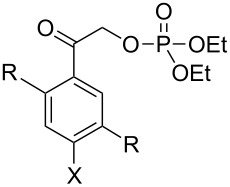	**4a** (R = H, X = OH)	MeOH	300	0.77 (87–96)	T^1^	Favorskii	[6,8,15,26,28–29]
	**4b** (R = H, X = OMe)	H_2_O, MeOH	300, 355	0.42^f^ (27^d^/90^f^)	S^1^	mixture	[28,36,57]
	**4c** (R = Me, X = H)	MeOH	313	0.71 (94)	nr	intact	[59]
benzoin 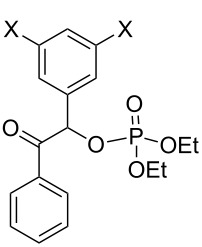	**2a** (X = H)	benzene	350	0.28 (72–79)	T^1^	benzofuran	[35,56]
	**2a** (X = H)	H_2_O/MeCN	350	0.37 (50^d^, 100)	T^1^	N/A^d^, benzofuran	[56,58,60]
	**2b** (X = OMe)	H_2_O	308, 350	N/A (55, 100)		benzofuran	[8,56,61]
coumarin-4-ylmethyl 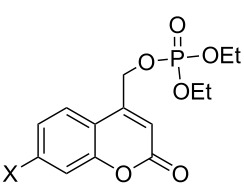	**3a** (X = OH)	MeOH	350	0.038 (<50)	S^1^	intact	[62–63]
	**3b** (X = OMe)	H_2_O/CH_3_CN, MeOH	333–350	0.04–0.08	S^1^	intact	[58,60]
	**3c** (X = NMe_2_)	H_2_O	389	0.0041 (N/A)	S^1^	intact	[62,64]
naphthylmethyl	**28** α	MeOH	300	0.08 (N/A)	S^1^	intact	[35,58]
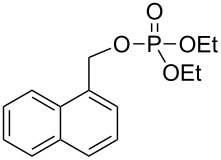	**29** β	MeOH	300	0.3 (N/A)	S^1^	intact	[35,58]
naphthylacetyl 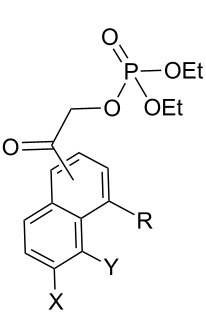	**10** (2,6) (X = OH, R, Y = H)	1% aq MeCN	350	0.031 (67)	T^1^	intact	this work
	**14a** (1,4) (X, Y = H, R = OH)	1% aq MeOH	350	0.028 (98)	T^1^	Favorskii	this work
	**14b** (1,4) (X ,Y = H, R = OMe)	1% aq MeOH	350	0.0076 (40)	T^1^	Favorskii	this work
	**27** (1,5) (X, R = H, Y = OH)	MeCN/H_2_O	300	nr (nr^g^)		intact	[19]
hydroxyindolinyl 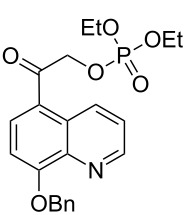	**24**	MeCN/H_2_O	350	24 × 10^−5^ (30–44)	S^1^	mixture	this work

^a^The ratio of H_2_O/MeCN was 1:1 unless stated otherwise. ^b^When the chromophore is unchanged upon photolysis, fate of the chromophore is noted as “intact”. When the chromophore rearranges by the photo-Favorskii migration or closure to a benzofuran group, the fate is noted as Favorskii or benzofuran, respectively. When complex photoreactions occur with the chromophore, it is noted as “mixture”. ^c^N/A = not available. ^d^Inorganic phosphate released in H_2_O ([56]), ^e^When internal return is included. ^f^In MeOH, ^g^nr = no reaction (leaving group was acetate).

Table 4 reveals that most chromophores either form complex mixtures of photoproducts complicating the isolation of pure, unprotected phosphates or they remain essentially unchanged (intact) and continue to absorb incident radiation, thus compromising the conversion to products and lengthening exposure of the sample to radiation. Three chromophores, benzoin, pHP, and now 1,4-hydroxynaphthylacetyl substrates cleanly undergo rearrangements that shift the absorption chromophore. Benzoins form benzofurans that, in fact, exacerbate the problem of light competition and fluorescence. Only pHP and 1,4-substituted naphthylacetyl **14** generate a less competitive chromophore allowing maximum conversion and quantitative chemical yield.

While pHP is highly efficient as a PPG (Φ’s approach 1.0) [15] the lower efficiency of 1,4-HNA (0.028) is competitive with many currently available PPGs. A measure often applied in biological decaging studies is the product of absorptivity and efficiency (ε_max_ × Φ) which is 232 for photolysis of 1,4-HNA DEP at 325 nm, competitive with PPGs listed in Table 4. By comparison, although its absorptivity is very low at 325 nm, pHP DEP has a much larger ε_max_ at 280 nm that gives a value of 6000 for excitation at 280 nm, an excitation wavelength that is biologically harmful [6,8–9].

## Conclusion

We have designed and tested several new candidates for near-UV absorbing photoprotecting groups. 1,4-HNA DEP (**14a**) and 1,4-MNA DEP (**14b**) upon irradiation at 350 nm in 1% aq MeOH quantitatively release diethyl phosphate concomitant with a photo-Favorskii rearrangement with quantum yields of 0.028 for 1,4-HNA DEP and 0.0076 for 1,4-MNA DEP. The lower quantum efficiency of 1,4-MNA in MeOH is fully in accord with a similar methylation of the hydroxy group of pHP DEP, emphasizing the role of the phenolic OH on the reaction and rearrangement [6,15,46,48]. In anhydrous, non hydroxylic media, the mechanism of the reaction changes from heterolysis and reverts to the more traditional photochemically induced homolysis mechanism, leading to more complicated product mixtures and poor conversions.

The heterolysis mechanism of the photo-Favorskii rearrangement and release of DEP is initiated at the triplet state of **14** and proceeds according to the more extensively characterized rearrangement for pHP DEP. Due to a more favorable excitation wavelength of 350 nm for 1,4-HNA (vs 280 nm for pHP), it is anticipated that the 4-hydroxy-1-acylnaphthyl PPG will find more favorable applications as a PPG not only for phosphates but many other acidic functional groups. Additional studies on 2PE activation of 1,4-HNA DEP at 650 nm are also in progress.

2,6-HNA DEP and 5,8-BQA DEP also release DEP but do not undergo the beneficial aryl ketone deconjugative rearrangement, instead follow a homolytic pathway. This results in lower yields, poorer efficiencies, and incomplete conversion for 2,6-HNA, and intractable mixtures of chromophoric byproducts and secondary oxidation reactions with O_2_, for 5,8-BQA. Neither chromophore is suitable for application as a PPG.

## Experimental

### Synthesis

The details on the synthesis of all new compounds can be found in the Supporting Information File 1. The synthetic procedures and spectral data for all new compounds are reported in Supporting Information File 1.

### Photochemistry

#### General methods

Photolyses were performed in a Rayonet RPR-100 photochemical reactor fitted with a merry-go-round apparatus and 16 × 350 nm (RPR 3500 Å) or 16 × 300 nm (RPR 3000 Å) lamps. The Rayonet reactor was turned on to warm up the lamps for 15 minutes prior to the irradiation. Samples were irradiated in NMR tubes or Pyrex test tubes at 40–45 °C in the presence of air unless indicated otherwise. The tubes were placed in a merry-go-round apparatus and the time of irradiation was recorded. Light output for the determination of quantum efficiencies was measured by using the potassium ferrioxalate method [65] under identical conditions used for the photolysis of the esters. Photolysis samples were analyzed for product formation and ester disappearance using ^1^H NMR and RP-HPLC methods. Dimethylformamide (DMF, 2 μL, 2.6 × 10^−2^ mmol) was used as an internal standard in ^1^H NMR analyses. For HPLC analyses the solvent was 70% CH_3_CN and 30% H_2_O unless otherwise noted. The detection wavelength varied depending on the ester analyzed as noted and the flow rate was 1.0 mL/min. Sample injections were performed in triplicate.

#### Photolysis of diethyl (2-(4-methoxynaphthalen-1-yl)-2-oxoethyl) phosphate (**14b**)

An NMR tube was charged with **14b** (16 mg, 0.045 mmol) along with CD_3_OD (0.95 mL) and DMF (2 µL) as an internal standard. The sample was irradiated at 350 nm and ^1^H NMR scans were collected after 0, 30, and 60 min of photolysis. The depletion of **14b** and the appearance of released diethyl phosphate were quantified from the integrations of the signals at δ 5.4 and 4.1 ppm, respectively. Results are shown in Table 1.

**1. Quantum efficiency determination.** A Pyrex tube was charged with **14b** (6.2 mg, 0.018 mmol) in methanol (5 mL) and water (50 µL) and the contents were mixed thoroughly. The sample was irradiated at 350 nm and sample aliquots (150 µL) were taken after 0, 15, 30, and 45 min. The aliquots were analyzed by HPLC with a mobile phase of 90% acetonitrile and 10% water and a detection wavelength of 319 nm. The depletion of **14b** was quantified from the peak areas of the signal at 3.5 min corresponding to the phosphate ester. The quantum efficiency was determined as described above. Results are shown in Table 1.

**2. Photoproduct analysis in aqueous methanol.** The photolysis mixture of **14b** from the previous experiment was concentrated under reduced pressure and the residue washed with water and extracted with ethyl acetate. The organic extract was concentrated under vacuum and the residue analyzed with ^1^H NMR (CD_3_OD) and FAB–MS. The data for the major product matched that of an authentic sample of methyl (4-methoxy-1-naphthyl)acetate (**26**).

**3. Photoproduct analysis in aqueous acetonitrile.** Diethyl (2-(4-methoxynaphthalen-1-yl)-2-oxoethyl) phosphate (**14b**, 6 mg, 0.017 mmol) was dissolved in acetonitrile (5 mL) containing water (50 µL) in a Pyrex tube and irradiated at 350 nm with 9–3500 Å bulbs. Sample aliquots were taken after 0, 45, and 90 min of photolysis and analyzed by HPLC. The detection wavelength was 319 nm. The phosphate ester had a retention time of 5.6 min. A new peak emerged at 6.3 min that grew in intensity throughout the photolysis. Co-injection with an authentic sample of 1-acetyl-4-methoxynaphthalene (**12**) confirmed the ketone as a photoproduct from the reaction.

**4. Diethyl (2-(4-methoxynaphthalen-1-yl)-2-oxoethyl) phosphate (14b): Dark reaction control experiment.** An NMR tube was charged with **14b** (ca. 5 mg), which was dissolved in methanol-*d*_4_ (<1 mL). The sample was heated to 46 °C in a warm water bath in the dark for 1 h. ^1^H NMR scans were taken before and after heating; no significant difference was observed in the NMR spectra. Also, **14a** was shown to be stable for 30 h in 10% aq MeCN at rt in the dark.

#### Photolysis of diethyl (2-(4-hydroxynaphthalen-1-yl)-2-oxoethyl) phosphate (**14a**)

An NMR tube was charged with **14a** (17 mg, 0.05 mmol) along with CD_3_OD (0.95 mL) and DMF (2 µL). The sample was irradiated at 350 nm and the percent conversion and deprotection % yield were determined as described above for **14b**. Results are shown in Table 1.

**1. Quantum efficiency determination in aqueous methanol.** The same general procedure and HPLC conditions were employed as described for **14b**. Amounts used: **14a** (7.6 mg, 0.022 mmol), methanol (5 mL), water (50 µL). The detection wavelength was 323 nm. The phosphate ester had a retention time of 3.1 min. Results are shown in Table 1.

**2. Quantum efficiency determination in aqueous acetonitrile.** A Pyrex tube was charged with **14a** (4.7 mg, 0.014 mmol), acetonitrile (5 mL) and water (50 µL). The sample was irradiated at 350 nm; aliquots (200 µL) were taken after 0, 2, 5, 7, and 10 min of photolysis and analyzed by HPLC on a C18 analytical column. The mobile phase contained 70% acetonitrile and 30% water, and the detection wavelength was 323 nm. The phosphate ester had a retention time of 3.5 min. The depletion of **14a** was accompanied by the appearance of four new peaks at 1.7, 2.3, 2.6, and 3.9 min, which were not identified. Results are shown in Table 2.

**3. Quantum efficiency determination in aqueous acetonitrile (degassed).** A Pyrex tube was charged with **14a** (5 mg, 0.015 mmol) along with acetonitrile (5 mL) and water (50 µL). The tube was fitted with a rubber septum and sparged with argon for 15 min. Sample aliquots (0.2 mL) were taken after 0, 2, 5, 7, and 10 min of photolysis using a needle syringe to avoid the introduction of air into the sample during irradiation. The aliquots were analyzed as described above. The percent conversion was 44% after 2 min of photolysis, thus the first two data points from a plot of mmol **14a** vs time were used to estimate the slope of the regression line for depletion of **14a** which led to an approximate value of 0.067 for the disappearance quantum efficiency (Table 2).

**4. Triplet sensitization experiment.** A quartz tube was charged with **14a** (2.0 mg, 0.0059 mmol) and benzophenone (20 mg, 0.11 mmol), and the contents were dissolved in acetonitrile (10 mL) to which was added water (100 µL) to make a 1% aqueous solution. The solution was degassed with argon and irradiated with 6-RPR 2540 Å bulbs. Sample aliquots (0.2 mL) were taken after 0, 2, 5, 7, and 10 min of photolysis and analyzed by HPLC on a C18 analytical column with a mobile phase containing 70% acetonitrile and 30% water. The detection wavelength was 323 nm. The benzophenone had a retention time of 6.5 min and remained constant throughout the photolysis. The phosphate ester had a retention time of 3.6 min and its depletion was accompanied by the appearance of three new peaks at 1.6, 1.9, and 4.4 min, which were not assigned. The quantum efficiency was determined as described above. Results are shown in Table 2.

**5. Photoproduct analysis in aqueous methanol.** A Pyrex tube was charged with **14a** (5 mg, 0.015 mmol), methanol (5 mL) and water (50 µL). The sample was irradiated at 350 nm for 15 min. After photolysis the solution changed from colorless to light yellow in appearance. The solvent was removed under reduced pressure and the remaining residue was dissolved in methylene chloride and washed with water. The organic extract was collected and the solvent concentrated to afford ca. 3 mg of an orange-colored residue. The residue was analyzed with ^1^H NMR in methanol-*d*_4_. Two signals were observed in the spectrum at δ 4.00 and 3.68 ppm, in a ratio of ca. 1:1.5, which were assigned to the methylene and methyl protons of methyl (4-hydroxy-1-naphthyl)acetate (**25**), based on the similar chemical shifts observed for methyl (4-methoxy-1-naphthyl)acetate (**26**).

**6. Photoproduct analysis in aqueous acetonitrile.** An NMR tube containing **14a** (ca. 5 mg) was charged with CD_3_CN (1 mL) and D_2_O (0.1 mL). The solution was mixed thoroughly and irradiated at 350 nm for 30 min. The ^1^H NMR spectrum contained a singlet at δ 2.66 ppm that was assigned to the methyl protons of 1-acetyl-4-hydroxynaphthalene (**15**). The presence of **15** was confirmed upon spiking with an authentic sample.

#### Photolysis of diethyl (2-(6-hydroxynaphthalen-2-yl)-2-oxoethyl) phosphate (**10**)

**Quantum efficiency determination.** A Pyrex tube was charged with **10** (2.2 mg, 0.0065 mmol) along with acetonitrile (5 mL) and water (50 µL). The solution was irradiated at 350 nm and sample aliquots (200 µL) were taken after 0, 2, 5, 7, and 10 min of photolysis. The aliquots were analyzed by HPLC. The detection wavelength was 315 nm. The phosphate ester had a retention time of 3.4 min and its depletion was accompanied by the appearance of signals at 1.8 and 4.0 min. The quantum efficiency was determined as described above and displayed in Scheme 5 (Φ_dis_= 0.031).

#### Photolysis of 4-[2-(6-hydroxy-2-naphthyl)-2-oxoethoxy]-4-oxobutan-1-aminium trifluoroacetate (2,6-HNA GABA)

**Photoproduct analysis.** A Pyrex tube was charged with 2,6-HNA GABA (19.5 mg, 0.068 mmol) along with acetonitrile (1.0 mL) and 50 mM TRIS buffer (9.0 mL, pH 7.3). The solution was photolyzed at 350 nm for 1 h, and sample aliquots of 50 µL were taken after 0, 30, and 60 min of photolysis. The aliquots were diluted with 150 µL of TRIS buffer and injected into an Econosphere C18 analytical column. The mobile phase contained 90% CH_3_CN with 0.1% TFA and 10% H_2_O. The flow rate was 1.0 mL/min and the detection wavelength was 244 nm. The depletion of 2,6-HNA GABA (retention time ca. 6 min) was accompanied by an increase in a peak with a retention time of ca. 3 min corresponding to the photoproduct(s). At the end of the photolysis, the solution contained an orange-colored precipitate. The organic soluble components were extracted with ethyl acetate, and the organic extract was washed with water, dried over magnesium sulfate, and the solvent removed under reduced pressure to afford ca. 6 mg of a yellow residue. The residue was dissolved in acetonitrile and injected into the HPLC column, resulting in a peak with a retention time of ca. 3 min. Co-injection of an authentic sample of 2-acetyl-6-hydroxynaphthalene (**6**) also produced the same peak. The residue was analyzed with ^1^H NMR (CDCl_3_) and found to contain the characteristic methyl singlet at δ 2.7 ppm corresponding to **6**, in addition to other unassignable peaks further downfield in the spectrum. TLC analysis with 2:1 hexane/ethyl acetate further suggested the presence of **6** as a photoproduct from the reaction.

**Fluorescence measurements.** A solution of 2,6-HNA GABA (0.042 mM in pH 7.3 TRIS buffer containing 1% acetonitrile) was placed in a quartz cell in a Carey Eclipse fluorescence spectrometer. Measurements were made under ambient conditions.

#### Photolysis of 2-(8-(benzyloxy)quinolin-5-yl)-2-oxoethyl diethyl phosphate (**24**)

An NMR tube was charged with **24** (5.0 mg, 0.01 mmol) dissolved in CD_3_CN (900 μL), D_2_O (100 μL), and DMF (2 μL, 2.6 × 10^−2^ mmol). The sample was irradiated without degassing with 16 × 300 nm or 16 × 350 nm lamps and ^1^H NMR spectra (16 scans) were collected after 0, 30, 60, 90, and 120 min of photolysis. The depletion of **24** and the appearance of released diethyl phosphate were quantified from the integrations of the methylene signals at δ 5.30 ppm and 3.89 ppm, respectively.

**1. Photolysis of 24 in the absence of oxygen.** Using the same general procedure, the photolysis was repeated under degassed conditions. In these experiments, photolysis samples were purged with argon for 30 min before photolysis. The results are shown in Table 3.

**2. Photoproduct analysis by ^1^H NMR.** An NMR tube was charged with **24** (10 mg, 0.02 mmol) which was dissolved in CD_3_CN (900 μL) and D_2_O (100 μL). The contents were mixed thoroughly and the sample irradiated with air or under Ar purged conditions with 16 × 300 or 350 nm lamps. ^1^H NMR spectra were collected after 60 min of photolysis and used to calculate the percent conversion. The depletion of **24** and the appearance of released diethyl phosphoric acid were determined from the NMR signals for the methylene protons of the phosphate ester and methylene hydrogens of diethyl phosphoric acid at δ 5.30 ppm and 3.88 ppm, respectively. The photolysis sample was spiked with an authentic sample of diethyl phosphoric acid synthesized from diethyl chlorophosphate [66]. A dramatic increase in the intensities of the new methylene and methyl signals at 3.88 and 1.19 ppm, respectively, confirmed the presence of diethyl phosphate.

**3. Control experiments with 24; stability in the absence of irradiation.** An NMR tube was charged with **24** (10.0 mg, 0.02 mmol) along with 900 μL of CD_3_CN and 100 μL of D_2_O. The contents were mixed thoroughly and warmed in a water bath at 40 °C in the dark for 2 h. ^1^H NMR analysis before and after the heating showed no significant change in the NMR spectrum. No significant change was observed when the sample was re-examined 48 h later.

## Supporting Information

File 1 Synthetic procedures and spectral data for all new compounds.
