# Supplementary material for: Photorelease of phosphates: Mild methods for protecting phosphate derivatives
Source: Beilstein J Org Chem. 2014 Aug 29;10:2038–54. doi: 10.3762/bjoc.10.212 (PMC4168878; doi:10.3762/bjoc.10.212)
Supplement: File 1 — Synthetic procedures and spectral data for all new compounds. [file Beilstein_J_Org_Chem-10-2038-s001.pdf]

Supporting Information

for

Photorelease of phosphates: Mild methods for protecting phosphate derivatives

Sanjeewa N. Senadheera[1,§], Abraham L. Yousef[1,‡]

and Richard S. Givens*[,1]

Address: [1]Department of Chemistry, University of Kansas, Lawrence, Kansas 66045, U.S.A.

[§]*Present address: Department of Medicinal Chemistry, University of Kansas, Lawrence, Kansas 66045, U.S.A.*

[‡]*Present address: Department of Chemistry, Sweet Briar College, Sweet Briar, VA 24595, U.S.A.*

Email: Richard S. Givens - givensr@ku.edu

*****Corresponding author

Synthetic procedures and spectral data for all new compounds

Contents

Materials and general methods	S3
Synthesis of the acetylnaphthylmethyl series, compounds **6–18**	S4–S12
Synthesis of the pHP-GABA derivative (2,6-HNA GABA)	S7–S8
Synthesis of the 5-acetylquinoline series, compounds **20–24**	S13–S16
Synthesis of photoproducts, compound **26**	S17
References	S18

General Methods: All compounds were prepared by literature procedures unless indicated otherwise. All reagents were purchased from commercial sources (Sigma Aldrich, St. Louis, MO, USA; Acros Organics, Thermo Fisher Scientific, NJ, USA; Alfa Aesar, Ward Hill, MA, USA, and Fisher Scientific, Pittsburg, PA, USA) and used without further purification unless otherwise noted. Reaction solvents were distilled prior to use. Acetic anhydride was refluxed over $CaCl_2$ for 3 days and distilled prior to use. Tetramethylammonium diethyl phosphate was prepared from diethyl phosphate and tetramethylammonium hydroxide [1]. ^{1}H NMR spectra were recorded on a Bruker 400 MHz instrument unless otherwise noted. Samples were dissolved in chloroform-*d* ($CDCl_3$), methanol-d_4 (CD_3OD), acetonitrile-d_3 (CD_3CN) or deuterium oxide-d_2 (D_2O) and chemical shifts are reported in parts-per-million, δ ppm. IR spectra were recorded on a Shimadzu FTIR-8400S spectrophotometer; results are reported in cm^{-1}. UV–vis spectra were recorded on a Cary 100 spectrophotometer (Varian, Inc., Palo Alto, CA, USA). pH values of solutions were measured using a Fisher Scientific pH 510 meter calibrated with certified Fisher buffer solutions of pH 4, 7, and 10. HPLC analyses were performed with a C18 Econosphere 250 × 4.6 mm analytical column (Altech Associates, Inc., Deerfield, IL, USA) connected to a Rainin dual pump system. Mass spectrometry was performed on a Sciex API-1 Plus quadrupole mass spectrometer with an electrospray ionization (ESI) source. Thin-layer and column chromatography were performed on precoated silica gel plates (Sorbent Technologies, Atlanta, GA, USA) and standard grade (32–63 μm) silica gel (Sorbent Technologies, Atlanta, GA, USA), respectively. Melting points were determined on a Thomas-Hoover melting point apparatus (Arthur H. Thomas Company, PA) and are uncorrected.

Synthesis of the Acetylnaphthylmethyl series:

2-acetyl-6-hydroxynaphthalene [2] (**6**) To a 25 mL rb flask was added 2-acetyl-6-methoxynaphthalene (**5**, 1.004 g, 5.0 mmol) along with potassium carbonate (49 mg, 0.35 mmol) and dry *N*-methyl-2-pyrrolidinone (3 mL). The contents were stirred for 5 min under a blanket of argon prior to the addition of thiophenol (0.770 mL, 7.5 mmol). The mixture was then heated up to 194 ^{o}C for 45 minutes with vigorous stirring. After the solution was allowed to cool to room temperature, methanol (25 mL) and triethylamine (1 mL) were added and the contents were transferred to a 100 mL rb flask. Hydrogen peroxide (1 mL, 30% solution) was added, and the solution was stirred for 10 minutes, then concentrated to a few milliliters under reduced pressure. The remaining liquid was diluted with 5% aqueous KOH and extracted with ether. The ethereal phase was discarded, and the aqueous portion was acidified with 6 M HCl (ca. pH 2) in an ice bath. The crude product was extracted with ether, and the ethereal phase was washed with brine, dried over $MgSO_4$, and evaporated under reduced pressure. Silica gel column chromatography (20:1 → 4:1 hexane:ethyl acetate) and collection of the appropriate fractions led to the isolation of 2-acetyl-6-hydroxynaphthalene (**6**) as a yellow solid. Yield: 675 mg, 72%; mp 170-172 ^{o}C, ^{1}H-NMR (400 MHz, $CDCl_3$) δ = 8.43 (s, 1H), 8.02 (dd, *J* = 8.7, 1.8 Hz, 1H), 7.90 (dd, *J* = 7.5, 2.1 Hz, 1H), 7.74 (d, *J* = 8.7 Hz, 1H), 7.22-7.20 (m, 2H), 5.87 (bs, 1H), 2.73 (s, 3H); ^{13}C NMR (400 MHz, CD_3OD) δ = 199.46, 158.38, 138.21, 131.94, 131.62, 130.96, 127.53, 126.56, 124.06, 119.37, 108.97, 25.52; IR (KBr, cm^{-1}) 3362, 3072, 2999, 1661, 1626, 1485, 1435, 1369, 1286, 1205.

2-acetyl-6-(*tert*-butyldimethylsilyloxy)naphthalene (**7**) A 50 mL rb flask was charged with *tert*-butyldimethylsilyl chloride (TBSCl, 503 mg, 3.3 mmol), 2-acetyl-6-hydroxynaphthalene (**6**, 551 mg, 3.0 mmol), and methylene chloride (10 mL). Triethylamine (418 µL, 3.0 mmol) and DBU (45 µL, 0.3 mmol) were added and the mixture was allowed to stir at room temperature for 28 h. The reaction mixture was then washed with cold 0.5 M HCl and the product extracted with methylene chloride. The organic extract was washed with saturated $NaHCO_3$, dried over $MgSO_4$, and the solvent evaporated under reduced pressure to obtain 2-acetyl-6-(*tert*-butyldimethylsilyloxy)naphthalene (**7**) as an off-white colored solid. Yield: 712 mg, 89%; ^{1}H-NMR (400 MHz, $CDCl_3$) δ = 8.42 (s, 1H), 8.01 (dd, *J* = 8.7, 1.7 Hz, 1H), 7.87 (d, *J* = 8.8 Hz, 1H), 7.75 (d, *J* = 8.7 Hz, 1H), 7.24 (d, *J* = 2.3 Hz, 1H), 7.16 (dd, *J* = 8.8, 2.4 Hz, 1H), 2.72 (s, 3H), 1.05 (s, 9H), 0.31 (s, 6H).

2-bromo-1-[6-(*tert*-butyldimethylsilyloxy)-2-naphthyl]ethanone (**8**) A flame dried 25 mL rb flask was charged with 2-acetyl-6-(*tert*-butyldimethylsilyloxy)naphthalene (**7**, 237 mg, 0.79 mmol) and dry chloroform (10 mL) under an argon atmosphere. Copper(II) bromide (401 mg, 1.8 mmol) was added along with ethyl acetate (10 mL), and the mixture was heated at reflux for 10 h. After cooling to room temperature, the contents were poured through a plug of silica gel and washed with copious amounts of methylene chloride. The solvent was removed under reduced pressure and the remaining crude solid was purified by silica gel column chromatography using 10:1 hexane:ethyl acetate as eluent to furnish 2-bromo-1-[6-(*tert*-butyldimethylsilyloxy)-2-naphthyl]ethanone (**8**) was obtained as a waxy solid. Yield: 256 mg, 96%. ^{1}H-NMR

(400 MHz, $CDCl_3$) δ 8.47 (s, 1H), 8.00 (dd, *J* = 8.7, 1.8 Hz, 1H), 7.91-7.87 (d, *J* = 8.8 Hz, 1H), 7.78 (d, *J* = 8.7 Hz, 1H), 7.24-7.23 (d, *J* = 2.1 Hz, 1H), 7.21-7.17 (dd, *J* = 8.8, 2.4 Hz, 1H), 4.58 (s, 2H), 1.05 (s, 9H), 0.30 (s, 6H).

2-bromo-1-(6-hydroxy-2-naphthyl)ethanone (**9**) A 100 mL rb flask was charged with 2-bromo-1-[6-(*tert*-butyldimethylsilyloxy)-2-naphthyl]ethanone (**8**, 240 mg, 0.63 mmol) and 30% aqueous methanol (10 mL). Methylene chloride (ca. 1-2 mL) was added as a co-solvent to help solubilize the starting material. Potassium bisulfate (226 mg, 1.66 mmol) was added and the contents were stirred at room temperature overnight. The reaction progress was monitored with TLC (2:1 hexane:ethyl acetate), which indicated a significant amount of starting material remaining after 12 h. Therefore an extra amount of potassium bisulfate (274 mg, 2.01 mmol) was added and the contents were stirred for an additional 24 h, for a total reaction time of 43 h. The solvent was removed under reduced pressure, and the remaining residue was dissolved in ethyl acetate and washed with water. The organic extract was dried over $MgSO_4$ and evaporated to provide the crude product. Silica gel column chromatography using a gradient eluent of 20:1 → 1:1 hexane:ethyl acetate afforded 2-bromo-1-(6-hydroxy-2-naphthyl)ethanone (**9**) as a bright yellow solid. Yield: 125 mg, 75%. Spectroscopic data are in strong agreement with the reported data [3].

Diethyl (2-(6-hydroxynaphthalen-2-yl)-2-oxoethyl) phosphate (**10**) A 50 mL rb flask was charged with 2-bromo-1-(6-hydroxy-2-naphthyl)ethanone (**9**, 86 mg, 0.32 mmol) and dry DMF (2-3 mL) under an argon atmosphere. Tetramethylammonium diethyl

phosphate (215 mg, 0.95 mmol) was added and the contents were heated up 55 ^{o}C for 2 h. After cooling to room temperature, the reaction mixture was washed with 5% H_2SO_4 and the product extracted with methylene chloride. The organic extract was dried over $MgSO_4$ and evaporated under reduced pressure. The remaining material was chromatographed on silica gel using 10:1 → 1:1 hexane:ethyl acetate as the eluent, to obtain diethyl (2-(6-hydroxynaphthalen-2-yl)-2-oxoethyl) phosphate (**10**) as a white solid. ^{1}H-NMR (400 MHz, CD_3OD) δ = 8.47 (s, 1H), 7.95 (m, 2H), 7.75 (d, J = 8.7 Hz, 1H), 7.20-7.18 (m, 2H), 5.54 (d, J = 11.0 Hz, 2H), 4.20 (p, J = 7.1 Hz, 4H), 1.39 (t, J = 7.0 Hz, 6H); ^{13}C-NMR (400 MHz, CD_3OD) δ = 192.74, 158.77, 138.56, 131.69, 130.09, 128.78, 127.43, 126.94, 123.54, 119.64, 109.04, 69.20 (d, J = 22.8 Hz), 64.80 (d, J = 23.6 Hz), 15.30 (d, J = 26.8 Hz); ^{31}P NMR (400 MHz, CD_3OD) δ 0.218; UV-Vis (CH_3CN) λ_{max} (ε) 213 (2.5 x 10^4), 242 (3.8 x 10^4), 260 (3.3 x 10^4), 315 (1.4 x 10^4); ES+ MS m/z (relative intensity): 339 (M+1, 100); exact mass calculated for $C_{16}H_{20}O_6P$ 339.0998, found 339.1000.

4-[2-(6-hydroxy-2-naphthyl)-2-oxoethoxy]-4-oxobutan-1-aminium trifluoroacetate (2,6-HNA-GABA)

The same general procedure was followed as for the synthesis of **10** with the exception that K_2CO_3 was used, the solvent was MeCN, and the reaction was carried out at room temperature in the dark for 20 h. Amounts used: 2-bromo-1-[6-(*tert*-butyldimethylsilyloxy)-2-naphthyl]ethanone (230 mg, 0.61 mmol), *N*-Boc-γ-aminobutyric acid (125 mg, 0.62 mmol), potassium carbonate (204 mg, 1.5 mmol), acetonitrile (20 mL); Yield: 91 mg, 38%. ^{1}H-NMR (400 MHz, $CDCl_3$) δ = 8.16 (s, 1H), 7.75-7.71 (m, 2H),

7.61-7.59 (d, J = 8.5 Hz, 1H), 7.20 (m, 2H), 5.38 (s, 2H), 4.95 (bs, 1H), 3.30 (m, 2H), 2.59 (t, 2H), 1.96 (p, 2H).

^{t}Boc deprotection

The general procedure for ^{t}Boc deprotection with TFA was that employed for the synthesis of pHP GABA [4]. Amounts used: 2-(6-hydroxynaphthalen-2-yl)-2-oxoethyl-4-(*tert*-butoxycarbonylamino) butanoate (91 mg, 0.23 mmol), trifluoroacetic acid (10 mL); Yield: 51 mg, 78%. ^{1}H-NMR (400 MHz, D_2O) $\delta =$ (s, 1H), 7.67-7.65 (d, J = 8.7 Hz, 1H), 7.56-7.49 (m, 2H), 7.03-7.01 (m, 2H), 5.31 (s, 2H), 3.02-2.98 (t, J = 7.4 Hz, 2H), 2.61-2.57 (t, J = 7.2 Hz, 2H), 1.97-1.90 (p, J = 7.2 Hz, 2H); ^{13}C-NMR (400 MHz, CD_3OD) $\delta =$ 193.28, 172.64, 155.67, 136.52, 130.63, 128.88, 126.58, 125.65, 125.54, 121.77, 117.78, 107.98, 65.32, 37.48, 29.13, 20.99. IR (KBr, cm^{-1}) 3426, 3068, 2940, 1747, 1680, 1629, 1511, 1178; UV-VIS in H_2O (λ_{max} (ε)): 213, 244, 261, 319 (1.0x10^4 M^{-1}cm^{-1}); FAB-MS m/z (relative intensity): 288 (M+, 44); exact mass calculated for $C_{16}H_{18}NO_4$ (M+) 288.1236, found 288.1226.

1-acetyl-4-methoxynaphthalene [5] (**12**) This compound was generated as reported previously [6].

2-bromo-1-(4-methoxy-1-naphthyl)ethanone [7]. (**13**) To a flame dried 50 mL rb flask was added 1-acetyl-4-methoxynaphthalene (**12**, 727 mg, 3.6 mmol) along with ethyl acetate (15 mL), chloroform (15 mL) and copper(II) bromide (1.79 g, 8.0 mmol). The reaction mixture was heated at reflux for 5 h. The reaction mixture was cooled to room temperature and filtered through a plug of silica gel with copious amounts of methylene

chloride. The crude product was purified with silica gel column chromatography, using 20:1 hexane:ethyl acetate as eluent, to afford 2-bromo-1-(4-methoxy-1-naphthyl)ethanone (**13**) as a yellow solid. Yield: 448 mg, 44%; mp 57-67 ^{o}C; ^{1}H-NMR (400 MHz, $CDCl_3$) δ = 8.97 (d, *J* = 8.7 Hz, 1H), 8.36 (d, *J* = 8.4 Hz, 1H), 8.07 (d, *J* = 8.3 Hz, 1H), 7.71-7.66 (m, 1H), 7.59-7.55 (m, 1H), 6.84 (d, *J* = 8.3 Hz, 1H), 4.57 (s, 2H), 4.11 (s, 3H); IR (KBr, cm^{-1}) 3072, 3007, 2984, 2941, 1647, 1569, 1510, 1465, 1456, 1386, 1255, 1238, 1207.

Diethyl (2-(4-methoxynaphthalen-1-yl)-2-oxoethyl) phosphate (**14b**) A flame dried 25 mL rb flask was charged with 2-bromo-1-(4-methoxy-1-naphthyl)ethanone (**13**, 374 mg, 1.3 mmol) and anhydrous dimethoxyethane (10 mL) under an argon atmosphere. Tetramethylammonium diethyl phosphate (400 mg, 1.8 mmol) was added and the reaction mixture was stirred at room temperature in the dark for 42 h, until no further change could be observed with TLC (2:1 hexane:ethyl acetate). The solvent was removed under reduced pressure, and the remaining residue was chromatographed on silica gel using a gradient eluent of 2:1 → 1:2 hexane:methylene chloride, then 100% methylene chloride followed by 2:1 methylene chloride:methanol to afford diethyl (2-(4-methoxynaphthalen-1-yl)-2-oxoethyl) phosphate (**14b**) as a white gummy solid. Yield: 429 mg, 92%. ^{1}H-NMR (400 MHz, $CDCl_3$) δ = 8.95 (d, *J* = 8.6 Hz, 1H), 8.33 (d, *J* = 8.4 Hz, 1H), 7.91 (d, *J* = 8.2 Hz, 1H), 7.67-7.63 (m, 1H), 7.60-7.53 (m, 1H), 6.79 (d, *J* = 8.2 Hz, 1H), 5.31 (d, *J* = 10.0 Hz, 2H), 4.24 (p, *J* = 7.2 Hz, 4H), 4.07 (s, 3H), 1.38 (t, *J* = 7.1 Hz, 6H); ^{13}C-NMR (400 MHz, $CDCl_3$) δ = 194.12, 160.30, 132.52, 131.23, 129.52, 126.56, 126.40, 126.30, 124.14, 122.64, 102.44, 69.70 (d, *J* = 21.2 Hz), 64.76 (d, *J* =

23.6 Hz), 56.34, 16.54 (d, J = 27.6 Hz); IR (Teflon, cm^{-1}) 3080, 2982, 2934, 1680, 1574, 1512, 1468, 1370, 1267, 1200, 1028, 980; UV-Vis (CH_3CN) λ_{max} (ε) 233 (3.4 x 10^4 $M^{-1}cm^{-1}$), 319 (1.2 x 10^4 $M^{-1}cm^{-1}$); FAB-MS: 353 (M+1); exact mass calculated for $C_{17}H_{22}O_6P$ 353.1154, found 353.1176.

1-acetyl-4-hydroxynaphthalene [8] (**15**) This compound was generated as reported previously [9].

1-acetyl-4-(*tert*-butyldimethylsilyloxy)naphthalene (**16**) A 50 mL rb flask was charged with TBSCl (216 mg, 1.43 mmol), 1-acetyl-4-hydroxynaphthalene (**15**, 222 mg, 1.19 mmol), and methylene chloride (ca. 10 mL). Triethylamine (166 µL, 1.2 mmol) and DBU (18 µL, 0.12 mmol) were added and the mixture was stirred at room temperature for 42 h, then washed with cold 0.5 M HCl and the product extracted with methylene chloride. The organic extract was washed with saturated $NaHCO_3$, dried over $MgSO_4$, and the solvent evaporated under reduced pressure to yield 1-acetyl-4-(*tert*-butyldimethylsilyloxy)naphthalene (**16**) as a yellow-beige solid. Yield: 307 mg, 86%. ^{1}H-NMR (400 MHz, $CDCl_3$) δ = 9.02 (d, J = 8.6 Hz, 1H), 8.28 (d, J = 8.4 Hz, 1H), 7.99 (d, J = 8.1 Hz), 7.66-7.62 (m, 1H), 7.56-7.54 (m, 1H), 6.86 (d, J = 8.0 Hz), 2.74 (s, 3H), 1.13 (s, 9H), 0.36 (s, 6H).

2-bromo-1-[4-(*tert*-butyldimethylsilyloxy)-1-naphthyl]ethanone (**17**) The same general procedure was followed as for the synthesis of 2-bromo-1-[6-tert-butyldimethylsilyloxy)-2-naphthyl)ethanone (**8**). The product **17** was isolated after

column chromatography (20:1 hexane:ethyl acetate) as a yellow crystalline solid. Yield: 330 mg, 89%. ^{1}H-NMR (400 MHz, $CDCl_3$) δ = 8.96 (d, *J* = 8.7 Hz, 1H), 8.30 (d, *J* = 8.3 Hz, 1H), 8.00 (d, *J* = 8.1 Hz, 1H), 7.70-7.66 (m, 1H), 7.59-7.55 (m, 1H), 6.87 (d, *J* = 8.1 Hz, 1H), 4.57 (s, 2H), 1.13 (s, 9H), 0.38 (s, 6H); ^{13}C-NMR (400 MHz, $CDCl_3$) δ = 193.02, 157.68, 133.57, 132.41, 131.43, 129.55, 128.73, 126.53, 124.69, 123.33, 110.93, 33.82, 26.20, 18.92, -3.77.

2-bromo-1-(4-hydroxy-1-naphthyl)ethanone (**18**) A 100 mL rb flask containing 2-bromo-1-[4-(*tert*-butyldimethylsilyloxy)-1-naphthyl]ethanone (**17**, 821 mg, 2.2 mmol) was charged with 30% aqueous methanol (20 mL) along with methylene chloride (ca. 3 mL) Potassium bisulfate ($KHSO_4$, 664 mg, 4.9 mmol) was added and the reaction mixture was stirred at room temperature overnight. The next day additional $KHSO_4$ was added (314 mg, 2.3 mmol) and the reaction progress monitored with TLC (2:1 hexane:ethyl acetate). After 40 h, the solvent was evaporated under reduced pressure and the remaining residue dissolved in ethyl acetate and washed with water. The organic extract was dried over $MgSO_4$ and evaporated to afford an orange residue. Silica gel column chromatography using 20:1 → 1:1 hexane:ethyl acetate provided, after recovery of unreacted starting material, 2-bromo-1-(4-hydroxy-1-naphthyl)ethanone (**18**). Yield: 239 mg, 59%. mp 168-170 ^{o}C; ^{1}H-NMR (400 MHz, $CDCl_3$) δ = 8.98 (d, *J* = 8.7 Hz, 1H), 8.30 (dd, *J* = 8.4, 0.7 Hz, 1H), 8.00 (d, *J* = 8.0 Hz, 1H), 7.73-7.69 (m, 1H), 7.62-7.58 (m, 1H), 6.86 (d, *J* = 8.0 Hz, 1H), 5.90 (bs, 1H), 4.57 (s, 2H); ^{13}C-NMR (500 MHz, $CDCl_3$) δ = 192.48, 156.50, 131.84, 129.59, 129.42, 126.39, 126.17, 126.12, 125.88, 121.92,

106.84, 33.10; IR (KBr, cm^{-1}) 3256, 1636, 1610, 1562, 1516, 1481, 1379, 1351, 1240, 1207; ES+ MS m/z (relative intensity) 265 (M+, 26), 267 (M+2, 23).

Diethyl (2-(4-hydroxynaphthalen-1-yl)-2-oxoethyl) phosphate (**14a**) A 50 mL rb flask was charged with 2-bromo-1-(4-hydroxy-1-naphthyl)ethanone (**18**, 230 mg, 0.87 mmol) and dry DMF (ca. 5-7 mL) under an argon atmosphere. Tetramethylammonium diethyl phosphate (626 mg, 2.75 mmol) was added and the contents were heated at 55 ^oC for 2 h. After cooling to room temperature, the reaction mixture was washed with 5% H_2SO_4 and the product extracted with methylene chloride. The organic extract was dried over $MgSO_4$ and evaporated under reduced pressure. The remaining material was chromatographed on silica gel using 10:1 → 1:1 hexane:ethyl acetate, then finally 100% ethyl acetate, to obtain diethyl (2-(4-hydroxynaphthalen-1-yl)-2-oxoethyl) phosphate (**14a**) as a white-pink colored solid. Yield: 91 mg, 31%; mp 154-156 ^oC; ^1H-NMR (400 MHz, CD_3OD) δ **=** 9.00 (d, *J* = 8.6 Hz, 1H), 8.33 (d, *J* = 8.4 Hz, 1H), 8.00 (d, *J* = 8.2 Hz, 1H), 7.65-7.60 (m, 1H), 7.54-7.50 (m, 1H), 6.87 (d, *J* = 8.2 Hz, 1H), 5.36 (d, *J* = 11.2 Hz, 2H), 4.24 (p, *J* = 7.1 Hz, 4H), 1.37 (t, *J* = 7.1 Hz, 6H); ^{13}C-NMR (400 MHz, CD_3OD) δ = 193.92 (d, *J* = 16.8 Hz), 159.65, 133.12, 132.36, 128.90, 125.65, 125.61, 125.47, 122.75, 122.01, 106.32, 69.68 (d, *J* = 22.8 Hz), 64.75 (d, *J* = 24.4 Hz), 15.40 (d, *J* = 26.8 Hz); ^{31}P NMR (CD_3OD) δ = 2.95; IR (KBr) 3421, 3094, 2989, 1668, 1576, 1520, 1385, 1232, 1246, 1059, 1032, 991, 976; UV-Vis (1% aq. CH_3CN) λ_{max} (ε) 235 (2.6×10^4 $M^{-1}cm^{-1}$), 325 (1.1×10^4 $M^{-1}cm^{-1}$); FAB-MS m/z (relative intensity) 339 (M+1, 50); exact mass calculated for $C_{16}H_{20}O_6P$ (M+1) 339.0998, found 339.0973.

Synthesis of the 5-Acetylquinoline series:

5-Acetyl-8-hydroxyquinoline (**20**)

5-Acetyl-8-hydroxyquinoline (**20**) was prepared from 8-hydroxyquinoline (**19**) according to the method described by Matsumura [10] with some modifications. The resulting crude product was recrystallized from hot water to afford 5-acetyl-8-hydroxyquinoline (**20**) as colorless hair-like needles (1.03 g, 5.51 mmol, 40% yield): mp 111-112 °C; ^{1}H NMR and IR data are in strong agreement with the reported data [11]. ^{13}C NMR (100 MHz, $CDCl_3$) δ = 199.5, 157.1, 148.6, 138.5, 136.7, 134.4, 127.6, 125.2, 124.6, 108.6, 29.2; UV/Vis (NH_4OAc, pH 7), λ_{max} (ε $Lmol^{-1}cm^{-1}$) 381 (3743), 301 (9345), 263 (15945), 240 (18465), 222 (13637); MS (ESI (+)) *m*/*z* calcd for $(C_{11}H_{10}NO_2 + H)^+$ 188.0711, found 188.0703.

1-(8-(Benzyloxy)quinolin-5-yl)ethanone (**21**)

The general procedure of Iwakuma *et al*. [12] was utilized with modifications. 5-Acetyl-8-hydroxyquinoline (**20**, 100 mg, 0.53 mmol) was dissolved in DMF (5 mL). K_2CO_3 (185 mg, 1.34 mmol) was added and the reaction mixture stirred for 30 min. Benzyl bromide (64 µL, 0.53 mmol) was slowly added dropwise. The reaction mixture was stirred for 24 h and concentrated, and water (5 mL) was added. The aqueous mixture was extracted with EtOAc (8 mL x 4), washed with brine and water, and dried over anhydrous $MgSO_4$. The solvent was evaporated, and the residue purified by flash chromatography with EtOAc:hexane (1:1) to afford 1-(8-(benzyloxy)quinolin-5-yl)ethanone (**21**) as a yellowish brown solid (300 mg, 1.08 mmol, 100% yield): mp 124-127 °C (dec); ^{1}H NMR ($CDCl_3$) δ = 9.42 (1H, dd, *J* = 8.8 Hz, 1.5 Hz), 8.96 (1H, dd, *J* = 3.8 Hz, 1.4 Hz), 7.96 (1H, d, *J* =

8.3 Hz), 7.51 (1H, q, *J* = 4.1 Hz), 7.47 (1H, d, *J* = 7.3 Hz), 6.94 (1H, d, *J* = 8.3 Hz), 5.47 (2H, s), 2.62 (3H, s); ^{13}C NMR (125 MHz, $CDCl_3$) δ = 199.3, 158, 149.6, 140.1, 136.2, 135.8, 132.5, 129, 128.3, 128.1, 127.7, 126.4, 123.7, 71.4, 29.1; IR (KBr, cm^{-1}) 3430 (br), 1653, 1560, 1502; UV/Vis (CH_3CN), λ_{max} (ε $Lmol^{-1}cm^{-1}$) 315 (6950), 240 (20100), 208 (14500); MS(ESI (+)) *m*/*z* calcd for $(C_{18}H_{15}NO_2 + H)^+$ 278.1181, found 278.1177.

2-(8-Benzyloxy)quinolin-5-yl)-2,2-dimethoxyethanol (**22**)

The target compound was synthesized according to the general method of Recuero *et al*. [13] with modifications. To a solution of KOH (248 mg, 4.43 mmol) in MeOH (4 mL), a solution of 1-(8-(benzyloxy)quinolin-5-yl)ethanone (**21**, 154 mg, 0.55) in MeOH (4 mL) was slowly added at 0 °C followed by iodobenzenediacetate (244 mg, 0.83 mmol) in small portions over one minute. The mixture was allowed to warm to room temperature and stirred for 24 h. The reaction mixture was concentrated, suspended in EtOAc and washed with water (10 mL x 2). The aqueous layer was extracted with EtOAc (5 mL x 5) and the combined organic layers were washed with brine and water, and dried over anhydrous $MgSO_4$. The solvent was evaporated, and the residue purified by flash chromatography with 1:1 EtOAc:hexane (1:1) to afford 2-(8-benzyloxy)quinolin-5-yl)-2,2-dimethoxyethanol (**22**) as a yellowish brown solid (168 mg, 0.50 mmol, 90% yield): mp 168-176 °C (dec); ^{1}H NMR ($CDCl_3$) δ = 8.91 (dd, 1H, 4.1 Hz, 1.1 Hz), 8.85 (1H, dd, 8.8 Hz, 1.6 Hz), 7.81 (1H, d, *J* = 8.3 Hz), 7.53 (2H, d, *J* = 7.2 Hz), 7.44 (1H, q, *J* = 4.1 Hz), 7.40-7.31 (3H, m), 6.98 (1H, d, *J* = 8.4 Hz), 5.42 (2H, s), 3.95 (2H, s), 3.27 (6H, s); ^{13}C NMR (125 MHz, $CDCl_3$) δ = 154.6, 148.7, 140.4, 136.8, 133.5, 129, 128.8, 128.1, 127.5, 126.9, 125.9, 121.6, 108.7, 102.7, 70.8, 64, 49; IR (KBr, cm^{-1}) 3412(br), 3313, 2934,

1508, 1458, 1385, 825; UV/Vis (CH_3CN), λ_{max} (ε $Lmol^{-1}cm^{-1}$) 311 (4450), 240 (25000), 202 (32200); MS (ESI (+)) *m/z* calcd for ($C_{20}H_{21}NO_4$ + H)$^+$ 340.1549, found 340.1539; calcd for ($C_{20}H_{21}NO_4$ + Na)$^+$ 362.1369, found 362.1383 .

1-(8-Benzyloxy)quinolin-5-yl)-2-hydroxyethanone (**23**)

2-(8-Benzyloxy)quinolin-5-yl)-2,2-dimethoxyethanol (**22**, 168 mg, 0.49 mmol) was dissolved in 50% CH_3COOH (10 mL) and 10% HCl (4 mL) was added. The resulting mixture was stirred for 12 h. The reaction mixture was made basic with solid $NaHCO_3$, and the aqueous layer extracted with EtOAc (10 mL x 5). The combined organic layers were washed with brine and water, and dried over anhydrous $MgSO_4$. The solvent was evaporated, and the residue was purified by flash chromatography with 1:1 EtOAc:hexane to afford 1-(8-benzyloxy)quinolin-5-yl)-2-hydroxyethanone (**23**) as a yellowish brown solid (142 mg, 0.48 mmol, 98% yield): mp 53-55 °C; ^{1}H NMR ($CDCl_3$) δ = 9.57 (1H, d, *J* = 8.6 Hz), 9.06 (1H, d, J = 4.1 Hz), 7.92 (1H, d, *J* = 8.2 Hz), 7.62 (1H, q, *J* = 5.9 Hz), 7.50 (1H, d, *J* = 7.3 Hz), 7.39-7.29 (3H, m), 7.03 (1H, d, *J* = 8.2 Hz), 5.53 (2H, s), 4.86 (2H, s); ^{13}C NMR (125 MHz, $CDCl_3$) δ = 198.5, 159.3, 150.1, 140.4, 135.9, 135.1, 131.6, 129.1, 128.3, 128.2, 127.3, 124.4, 122.3, 108.2, 71.4, 65.8; IR (KBr, cm^{-1}) 3431 (br), 3277, 2924, 1670, 1601, 1564, 1506, 1385, 1315, 794; UV/Vis (CH_3CN), λ_{max} (ε $Lmol^{-1}cm^{-1}$) 322 (6230), 240 (17100); MS (ESI (+)) *m/z* calcd for ($C_{18}H_{15}NO_3$ + H)$^+$ 294.1130, found 294.1130; calcd for ($C_{18}H_{15}NO_3$ + Na)$^+$ 316.0950, found 316.0952.

2-(8-(benzyloxy)quinolin-5-yl)-2-oxoethyl diethyl phosphate (**24**)

The general method of Ma *et al*. [14] was followed with modifications. Under an Ar atmosphere, 1-(8-benzyloxy)quinolin-5-yl)-2-hydroxyethanone (**23,** 159 mg, 0.54 mmol) was dissolved in pyridine (5 mL). Diethyl chlorophosphate (166 μL, 1.15 mmol) was slowly added dropwise at -5 °C. The reaction mixture was allowed to warm to room temperature and stirred for 24 h. EtOAc (10 mL) and water (10 mL) were added to the reaction mixture and the EtOAc layer separated. The aqueous layer was extracted with EtOAc (10 mL x 3) and the combined organic layers were washed with brine and water, and dried over anhydrous $MgSO_4$. The solvent was evaporated, and the residue was purified by flash chromatography with a gradient solvent system of 100% hexane, 1:1 EtOAc:hexane, 2:1 EtOAc:hexane, 3:1 EtOAc:hexane, 100% EtOAc, and 10% MeOH in EtOAc to afford 2-(8-(benzyloxy)quinolin-5-yl)-2-oxoethyl diethyl phosphate (**24**) as a yellowish brown solid (70 mg, 0.16 mmol, 30% yield): mp 98-101 °C; ^{1}H NMR ($CDCl_3$) δ = 9.37 (1H, d, *J* = 8.7 Hz), 9.01 (1H, d, *J* = 3.2 Hz), 7.88 (1H, d, *J* = 8.3 Hz), 7.56 (1H, q, *J* = 3.7 Hz), 7.48 (2H, d, *J* = 7.5 Hz), 7.37-7.29 (3H, m), 6.99 (1H, d, *J* = 8.3), 5.50 (2H, s), 5.24 (2H, d, *J* = 10.2), 4.22-4.15 (4H, m), 1.35-1.31 (6H, m); ^{31}P NMR ($CDCl_3$) δ ppm -0.13; ^{13}C NMR (100 MHz, $CDCl_3$) δ = 193.1, 158.7, 150, 140.4, 135.9, 134.9, 131.2, 129, 128.4, 128.2, 127.2, 124, 123.1, 107.9, 71.2, 69.2, 64.5, 16.3-16.2; IR (KBr, cm^{-1}) 3416(br), 2984, 1686, 1599, 1560, 1502; UV/Vis (CH_3CN/H_2O), λ_{max} (ε $Lmol^{-1}cm^{-1}$) 321 (8370), 240 (20100), 208 (13400); MS (ESI (+)) *m/z* calcd for $(C_{22}H_{24}NO_6P + H)^+$ 430.1419, found 430.1414.

Synthesis of photoproducts:

Methyl (4-methoxy-1-naphthyl)acetate [15] (**26**) A 50 mL rb flask was flame dried and charged with 1-methoxynaphthalene (**11**, 5.0 mL, 35 mmol), ferric oxide (12 mg, 0.075 mmol), and methyl bromoacetate (1.6 mL, 17 mmol). The contents were heated to 177 ^{o}C. After 41 h, the temperature had increased to 190 ^{o}C and TLC (2:1 hexane:ethyl acetate) indicated the presence of one or more fluorescent products from the reaction. The reaction mixture was cooled to room temperature, then poured through a silica gel column with 10:1, 2:1 and finally 1:1 hexane:ethyl acetate to afford a brown, oily liquid (ca. 3.15 g). The crude liquid was further chromatographed on silica gel with 20:1 → 10:1 hexane:ethyl acetate. Two major fractions were isolated from the separation, the first of which was recovered 1-methoxynaphthalene (2.29 g), followed by an orange colored oil (309 mg). The oil was chromatographed on silica gel with 20:1 hexane:ethyl acetate, providing methyl (4-methoxy-1-naphthyl)acetate (**26**, 140 mg) as a brown-yellow oil. Yield: 140 mg, < 10%. Further purification of the oil was effected upon sublimation at ca. 170 ^{o}C. ^{1}H-NMR (400 MHz, $CDCl_3$) δ = 8.34 (dd, J = 8.1, 1.1 Hz, 1H), 7.94 (d, J = 8.5 Hz, 1H), 7.60-7.56 (m, 1H), 7.52 (m, 1H), 7.34 (d, J = 7.8 Hz, 1H), 6.80-6.78 (d, J = 7.8 Hz, 1H), 4.02 (s, 5H), 3.70 (s, 3H); ^{13}C-NMR (500 MHz, $CDCl_3$) δ = 172.49, 155.21, 132.87, 127.98, 126.91, 125.13, 123.61, 122.66, 122.51, 116.02, 103.33, 55.51, 52.12, 38.64; UV-Vis (CH_3CN) λ_{max} (ε) 212, 234, 297 (7.9 x 10^{3} $M^{-1}cm^{-1}$); FAB-MS m/z (relative intensity) 230 (M+, 56).

References:

1. Zwierzak, A.; Kluba, M. *Tetrahedron*, **1971***, 27*, 3163-3170.
2. Bisanz, T.; Bukowska, M. *Roczniki Chemii*, **1974***, 48*, 777-786.
3. Thirunarayanan, G.; Vanangamudi, G.; Sathiyendran, V.; Ravi, K. *Indian J. Chem*. **2011**, *50B*, 593 - 604.
4. Givens, R. S.; Weber, J. F. W.; Jung, A. H.; Park, C.-H. In *Methods in Enzymology*, Marriott, G., Ed.; Academic Press: New York, **1998**, *291,* pp. 1 - 29.
5. Dixon, E. A.; Fischer, A.; Robinson, F. P. *Can. J. Chem.* **1981***, 59*, 2629-2641.
6. Deutsch, J.; Prescott, H. A.; Muller, D.; Kemnitz, E.; Lieske, H. *J. Catal*. **2005**, *231*, 269-278.
7. Jacobs, T. L.; Winstein, S.; Ralls, J. W.; Robson, J. H.; Henderson, R. B. *et al*. *J. Org. Chem.* **1946***,11*, 21-26.
8. Bisanz, T.; Bukowska, M. *Roczniki Chemii*, **1974***, 48*, 777-786.
9. Lukeman, M.; Veale, D.; Wan, P.; Munasinghe, V. R. N.; Corrie, J. E. T. *Can. J. Chem.* **2004***, 82*, 240-253.
10. Matsumura, K. *J. Am. Chem. Soc*. **1930**, *52*, 4433-4436
11. Mohrle, H.; Schaltenbrand, R. *Pharmazie*, **1985**, *40*, 307-311.
12. Iwakuma, T.; Tsunashima, A.; Ikezawa, K.; Takaiti, O. *Eur. Pat. Appl*. **1985**, EP 147719 A2 19850710.
13. Recuero, V.; de Gonzalo, G.; Brieva, R.; Gotor, V. *Eur. J. Org. Chem*. **2006**, *18*, 4224-4230.
14. Ma, C.; Kwok, W. M.; Chan, W. S.; Zuo, P.; Kan, J. T. W.; Toy, P. H.; Phillips, D. L. *J. Am. Chem.Soc*. **2005**, *127*, 1463-1472.
15. Gielen, H.; Alonso-Alija, C.; Hendrix, M.; Niewöhner, U.; Schauss, D. *Tetrahedron Lett.* **2002***, 43*, 419 - 421.
